# Human FASTK preferentially binds single‐stranded and G‐rich RNA

**DOI:** 10.1111/febs.70155

**Published:** 2025-07-01

**Authors:** Daria M. Dawidziak, Dawid A. Dzadz, Mikołaj I. Kuska, Madhuri Kanavalli, Maria M. Klimecka, Matthew Merski, Katarzyna J. Bandyra, Maria W. Górna

**Affiliations:** ^1^ Structural Biology Group, Faculty of Chemistry, Biological and Chemical Research Centre University of Warsaw Poland

**Keywords:** FASTK, G‐rich RNA, RNA degradation, RNA‐binding proteins

## Abstract

Fas‐activated serine/threonine kinase (FASTK) is the founding member of the FASTKD protein family, which was shown to regulate the fate of mRNA molecules on multiple levels. The mitochondrial variant of FASTK co‐localizes with mitochondrial RNA granules and regulates the degradation of mitochondrial mRNAs, whereas the cytoplasmic and nuclear forms of FASTK are involved in the regulation of alternative splicing, cytoplasmic RNA granule formation, and mRNA translation. Despite these multiple roles of FASTK in mRNA biology, the exact rules of RNA recognition by this protein remained undetermined. Here, we demonstrate direct RNA binding by purified human FASTK and show its preference for single‐stranded G‐rich oligonucleotides, including those with a tendency to form RNA G‐quadruplexes. Addition of FASTK alone was sufficient to achieve protection of mitochondrial mRNAs from degradation by the degradosome. Structural characterization by SAXS (Small‐Angle X‐ray Scattering) showed that FASTK in solution is a monomer with an extended conformation. Point mutagenesis studies supported the structural predictions of an exposed RNA‐binding interface in the central helical region, preceded by a smaller, flexibly attached helical N‐terminal domain. We provide the first such extensive *in vitro* characterization of the RNA binding properties for a representative of the FASTKD protein family and suggest how these intrinsic properties may underlie FASTK function in mRNA metabolism.

## Graphical abstract

Although FASTK is known to play a role in mRNA biology, the mechanism through which it recognizes RNA has yet to be unraveled. Here, we used purified human FASTK to characterize its RNA‐binding properties *in vitro*. We found that FASTK prefers ssRNA oligonucleotides containing guanines with the potential to form G‐quadruplexes and binds robustly to any longer RNA such as mitochondrial mRNA. Moreover, the addition of FASTK alone could protect RNA from degradation by the degradosome. Structural characterization using SAXS data and predicted models verified by mutagenesis experiments identified an RNA‐binding interface in the central region of FASTK. Created in BioRender. Gorna, M. (2025) https://BioRender.com/mxobj8k

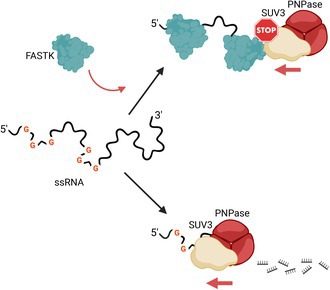

**Abbreviations**
ANOVA: analysis of variance
ATP: adenosine triphosphate
CDS: coding sequence
COX2: cytochrome c oxidase subunit 2
DMSO: dimethyl sulfoxide
dsRNA: double‐stranded ribonucleic acid
DTT: dithiothreitol
EDTA: ethylenediaminetetraacetic acid
EMSA: electrophoretic mobility shift assay
FASTK: Fas‐activated serine/threonine kinase
G4: G‐quadruplex
GST: Glutathione S‐transferase
IMAC: immobilized metal affinity chromatography
KD: dissociation constant
LC–MS: liquid chromatography coupled with mass spectrometry
MALS: multi‐angle light scattering
MBP: myelin basic protein
MRG: mitochondrial RNA granule
MST: microscale thermophoresis
ND6: NADH dehydrogenase subunit 6
PAGE: polyacrylamide gel electrophoresis
PBS: phosphate‐buffered saline
PMSF: phenylmethanesulfonylfluoride
RAP: RNA‐binding domain abundant in Apicomplexans
RBP: RNA‐binding protein
SAXS: Small‐Angle X‐ray Scattering
SEC: Size‐Exclusion Chromatography
SELEX: Systematic Evolution of Ligands by Exponential enrichment
ssRNA: single‐stranded ribonucleic acid
SUMO: Small Ubiquitin‐like Modifier
TCEP: tris(2‐carboxyethyl)phosphine
TERRA: telomeric repeat–containing RNA
UTR: untranslated region
β‐Me: β‐mercaptoethanol

## Introduction

The Fas‐activated serine/threonine kinase domain‐containing (FASTKD) family is one of the largest protein families involved in the regulation of mitochondrially encoded genes. The six human members of this family often show distinct, or even contradictory, influences on certain mitochondrial transcripts and localize to different mitochondrial compartments [1]. The eponymous FASTK [2] was demonstrated to co‐localize with mitochondrial RNA granules (MRGs) shown to be associated with posttranscriptional regulation, expression, and degradation of mt‐mRNAs [3, 4, 5]. In addition, FASTK has an alternative, non‐mitochondrial isoform implicated in the regulation of cell death and autoimmune disorders [1, 2, 6, 7, 8, 9]. FASTK was first discovered as a partner and a putative kinase of T‐cell‐restricted intracellular antigen‐1 (TIA‐1) [10], but this activity was questioned since the key residues in its then‐proposed active site are not preserved across the family [11].

**Fig. 1:**
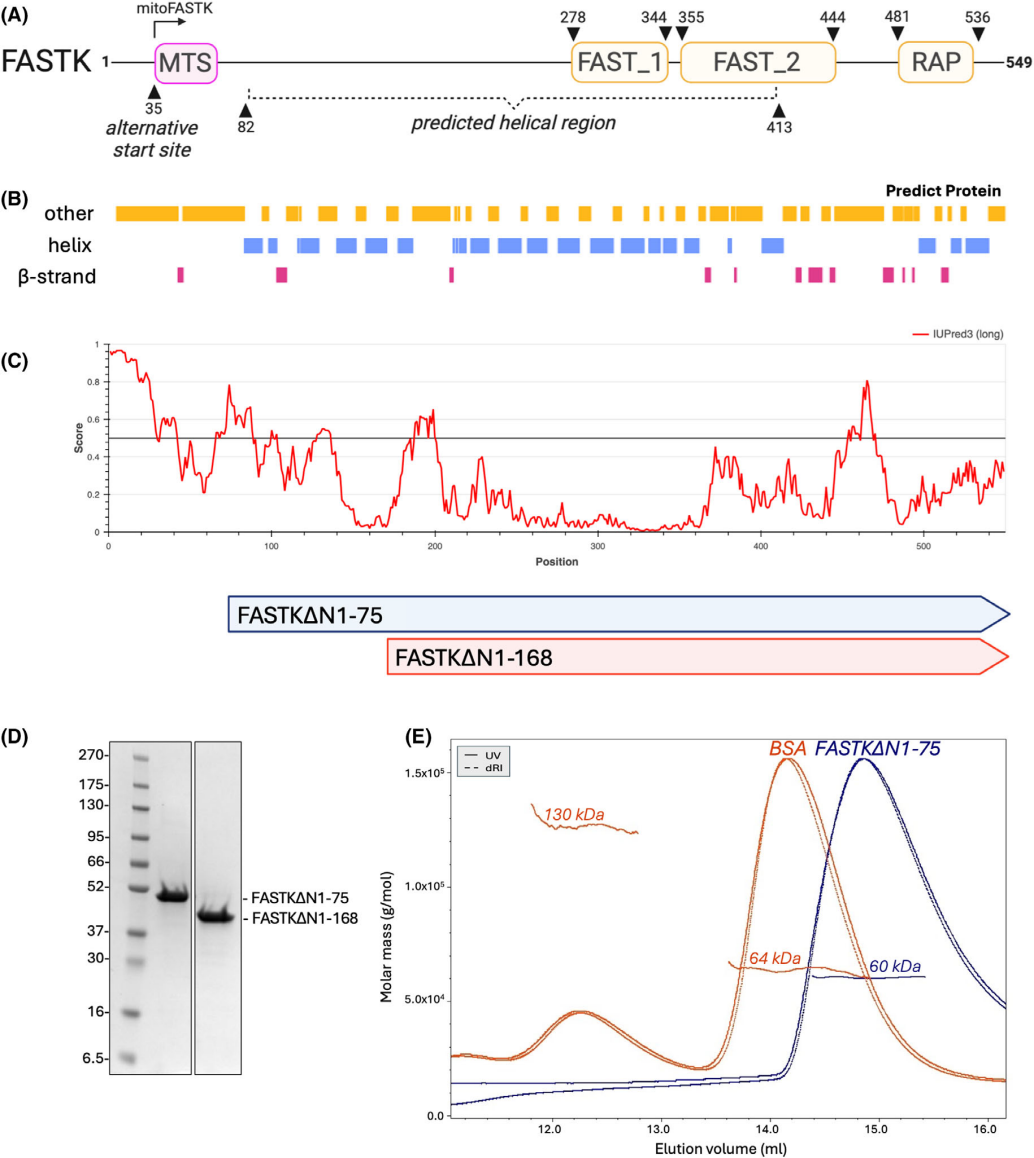
Analysis of FASTK secondary structure predictions, design of purification strategy, and analysis of FASTK oligomeric state. (A) Schematic representation of the structure of human FASTK protein. FAST_1 (FAST kinase‐like protein subdomain 1), FAST_2 (FAST kinase‐like protein subdomain 2), and RAP (RNA‐binding domain abundant in Apicomplexans) domains are indicated. MTS (mitochondrial targeting signal) indicates the start site of an alternative translational form of FASTK residing in mitochondria. (B) Diagram showing results of secondary structure predictions done with Predict Protein [23, 26]—yellow boxes represent regions with unknown secondary structure, purple and pink represent helices and beta‐strands, respectively. (C) Plot shows results of disordered regions predictions performed with IUPred3 [24], where a score between 0 and 1 corresponds to the probability of the given residue being part of a disordered region. Panel below shows amino acids range of FASTK constructs—FASTK∆N1‐75 and FASTK∆N1‐168. (D) Representative SDS/PAGE gel (*n* = 3) showing samples of two FASTK constructs purified according to the developed purification procedure. (E) SEC‐MALS analysis of the purified FASTK∆N1‐75 (blue trace) and BSA (red trace). Curves show the UV absorbance and refractive index signal. Horizontal lines show the calculated masses of the eluting components. Domain boundaries were assigned based on the Pfam database in InterPro web suite [25].

FASTKD family members share the same overall architecture: an N‐terminal mitochondrial targeting signal (MTS) followed by a region predicted to be mostly helical and containing three conserved sequence domains called FAST_1 (FAST kinase‐like protein subdomain 1), FAST_2 (FAST kinase‐like protein subdomain 2) and RAP (RNA‐binding domain abundant in Apicomplexans) (Fig. 1A) [12, 13, 14, 15]. Likely due to the presence of disordered regions impeding crystallization, only the structure of a FASTKD4 fragment containing these three sequence domains has been determined experimentally [16]. The RAP domain shows similarities to PD‐(D/E)XK nucleases and is necessary for the functions of FASTKD proteins in RNA metabolism [14, 16, 17]. So far, only FASTKD5 has been confirmed as a nuclease *in vitro* [18], whereas the other human paralogs seem to lack the necessary catalytic residues, and in these cases, RAP is expected to contribute to RNA binding [16].

The maturation of mitochondrial transcripts is executed mostly by removal of flanking tRNAs, according to the tRNA punctuation model; however, there are four transcripts which are not subject to this model [19]. One of them is ND6 mRNA encoding subunit 6 of the NADH dehydrogenase complex (complex I), which exists at steady state as a mature form of 1.0–1.1 kb [19]. FASTK was shown to be particularly important for maturation of this transcript and activity of the whole NADH dehydrogenase complex [2]. The skeletal and cardiac muscles from mice with disrupted FASTK gene displayed ~60% decrease in NADH dehydrogenase activity. Accordingly, levels of ND6 mRNA were reduced in MEFs isolated from FASTK‐depleted mice, suggesting that FASTK controls expression of this mRNA [2]. Indeed, RNA‐IP (co‐immunoprecipitation of RNA bound to the captured mitoFASTK‐HA) revealed that FASTK binds both ND6 mRNA and its precursors. Furthermore, it has been shown that human FASTK cooperates with the mitochondrial degradosome proteins—polynucleotide phosphorylase PNPase and helicase Suv3p—to generate mature ND6 mRNA by preventing its CDS and 3′UTR from degradation by the degradosome [2].

The ND6 gene is the only gene transcribed using the L‐strand of the mitochondrial genome, resulting in an mRNA encoded by the H‐strand and highly abundant in guanines. Such G‐rich L‐strand transcripts can theoretically form non‐canonical elements called G‐quadruplexes (G4s). ND6 has a predicted propensity to form G‐quadruplexes [20] and such G4‐containing RNAs might undergo degradation according to a dedicated surveillance mechanism employing GRSF1, which promotes melting of G4 structures and facilitates degradosome‐mediated decay. Interestingly, FASTK was shown to co‐localize with GRSF1 in mitochondria, but as an inhibitor of the degradosome, FASTK is expected to have an opposite effect to GRSF1 on G4‐containing RNA. FASTK was observed to bind along ND6 with enrichment in certain sections of this transcript [2], but the exact sequence motifs or other features of RNA recognized by FASTK have not been determined yet. In addition, it is not yet clear how RNA binding by FASTK outside the mitochondria contributes to its other roles in cell survival, apoptosis, and regulation of the immune system [6, 7, 8, 9, 21, 22].

Here, we aim to demonstrate and characterize RNA binding by human FASTK using highly purified recombinant protein and a range of RNAs. We compared sequences, secondary structures, and the nucleotide content of bound RNAs to describe the preference of FASTK. Our SAXS‐derived experimental structural information, supported by machine learning methods, provides hints about the RNA‐binding interface of FASTK.

## Results

### The design and optimization of recombinant FASTK purification strategy

The insoluble nature of bacterially expressed FASTK has been previously reported and has so far hindered research aiming at solving its 3D structure and explaining the basis of its interactions with RNA molecules [1]. We relied on secondary structure predictions [23, 24, 25, 26] combined with the Udwary–Merski algorithm (UMA) [27] to predict the location of structured and linker regions in the FASTK protein sequence (Fig. 1B,C) and generated two expression constructs (FASTK∆N1‐75 and FASTK∆N1‐168) which resulted in highly stable and soluble recombinant proteins (Fig. 1D). The purification conditions were optimized, and four crucial modifications were applied in order to increase protein solubility and purity. (i) The purification buffer composition was optimized based on protein melting temperatures using a thermal shift assay in the presence of additives from two commercial screens (Fig. S1). Buffer pH was adjusted to 8.5, and ammonium sulfate was added throughout to increase protein stability. (ii) 10 mm EDTA was added to the dialysis buffer during the cleavage of His6‐SUMO tag after IMAC HisTrap column purification to chelate Nickel ions and possibly prevent protein aggregation. (iii) The concentration of NaCl in the lysis buffer was increased to 500 mm, and an additional step of polyethyleneimine (PEI) precipitation was added to the procedure to remove nucleic acid contaminations. (iv) An additional step of ion exchange chromatography yielded pure protein freed from other protein contaminants.

The molecular weight and oligomeric state of the recombinant FASTK∆N1‐75 protein were confirmed using size‐exclusion chromatography (SEC) coupled with multi‐angle light scattering (MALS) (Fig. 1E). When injected at ~94 μm concentration, the protein eluted as a single peak with a calculated molecular weight (MW) of 60 kDa, close to that of a monomer (53 kDa as the expected MW from sequence), confirming that the FASTK construct lacking the N‐terminal unstructured region is a monomer in the solution.

To address the putative but disputed role of FASTK as a kinase of TIA‐1 [10, 11], FASTK∆N1‐75 kinase activity was assayed *in vitro* in the presence of recombinant TIA1‐RRM23 and [γ^32^P]ATP as substrates, but no phosphorylation activity was detected (Fig. S2). Very slight activity was observed in the gel area of the FASTK∆N1‐75 construct itself, suggesting some residual modification or non‐covalent binding between FASTK∆N1‐75 and [γ^32^P]ATP that might not necessarily be specific. This confirmed that recombinant FASTK∆N1‐75 does not have protein kinase activity under these conditions and suggests that regulation of TIA‐1 function by FASTK is the result of a different mechanism.

### FASTK prefers single‐stranded and G‐rich RNA

In our studies on the RNA‐binding specificity of FASTK, we first attempted to identify the preferred sequence motifs via SELEX [28] using GST‐tagged FASTK and a random library of short RNA oligonucleotides. This failed to enrich any high‐confidence sequence motifs bound by FASTK. Due to visible dimerization of amplified RNA oligonucleotides, two rounds of SELEX selection were employed, leading to the identification of three statistically significant RNA sequence motifs (Fig. S3), two of which happened to be mutually complementary single‐stranded RNAs. The biological significance of the discovered motifs was, however, questionable due to their relatively low abundance (occurring only 680, 227, and 84 times out of approximately 216 000 molecules sequenced). The failure of the SELEX assay suggests a lack of a strong sequence preference for FASTK, since the same library has yielded, before, a specific recognition motif for a different protein [29]. The SELEX library represented random 20mers (expected to include naturally occurring targets of FASTK), which is typically a sufficient length for a sequence‐specific protein. However, we could not exclude that longer oligonucleotides would be needed, for example, to form a secondary structure recognized by FASTK, or that multiple motifs should be spaced further apart, like in the case of ZAP requiring at least 15 CpG [30].

**Fig. 2:**
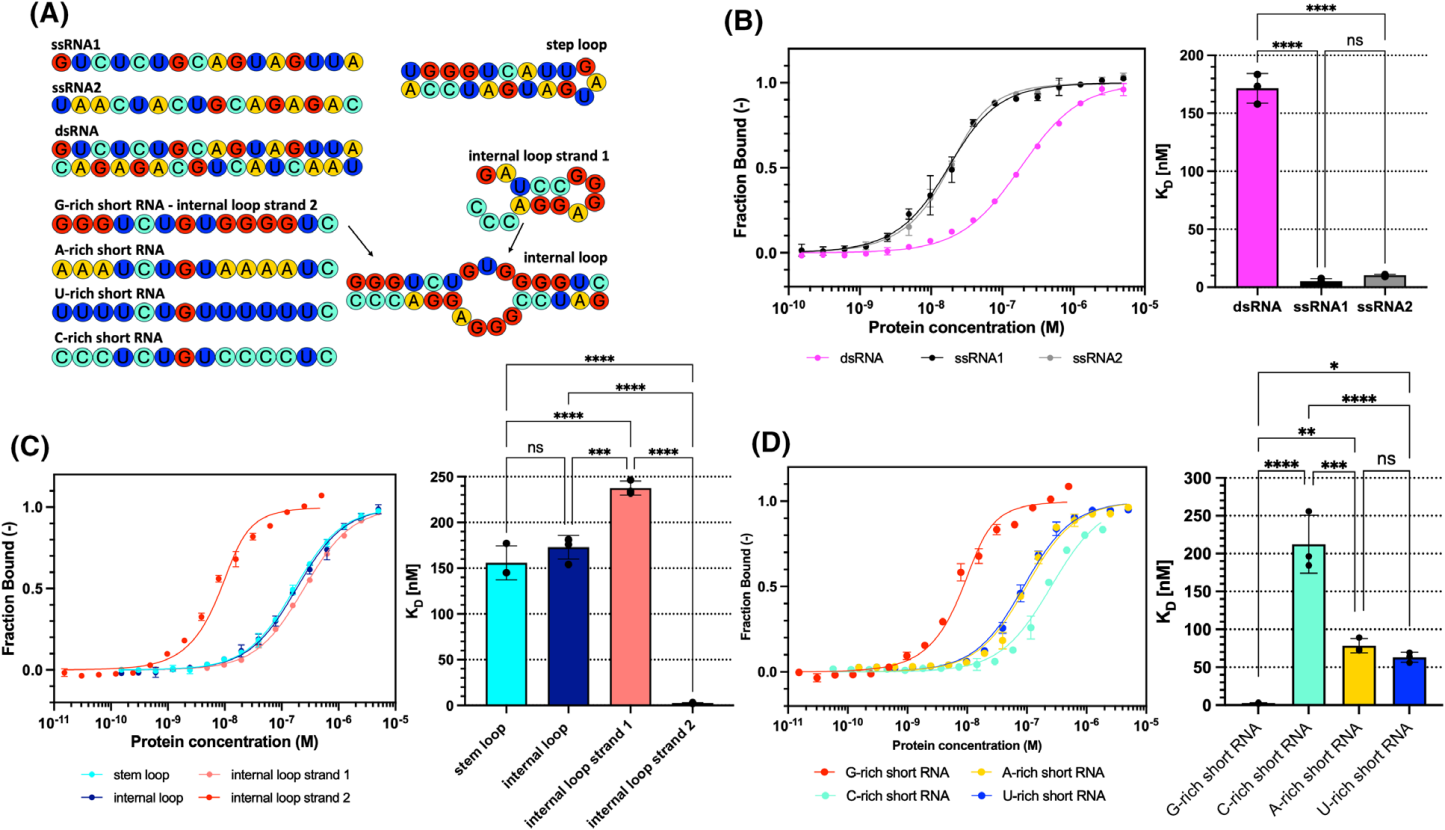
Microscale thermophoresis analysis of FASTK∆N1‐75 binding to structured, single‐ and double‐stranded RNAs. (A) Schematic representation of RNA oligonucleotides tested in binding assays. Binding of double‐stranded and single‐stranded RNA oligonucleotides (B), loop forming RNA oligonucleotides (C) and short RNA oligonucleotides (D) by FASTK∆N1‐75 was determined by microscale thermophoresis. The binding curves were plotted as a bound fraction of RNA against a protein concentration. Each measurement point was derived from three technical replicates, and error bars represent the SD. Statistical significance of pairwise comparisons was calculated with Tukey's multiple comparisons tests for an ordinary one‐way analysis of variance (ANOVA): *P* > 0.05 (ns); *P* ≤ 0.05 (*); *P* ≤ 0.01 (**); *P* ≤ 0.001 (***); *P* ≤ 0.0001 (****).

To further analyze the putative RNA targets of FASTK suggested by the best SELEX hits, we performed *in vitro* binding assays with synthetic oligonucleotides (Table S1) using microscale thermophoresis and native gel electrophoresis. We tested two ssRNAs initially selected by SELEX, a dsRNA formed by these two, and three other structural motifs such as an internal loop and a stem loop (Fig. 2A). The comparison of FASTK binding measurements (Fig. 2B,C) showed that the *K*_*D*_ values for loop forming, or double‐stranded oligonucleotides were notably weaker (155.8–237.6 nm) than for single‐stranded RNAs (2.7–10.3 nm). Moreover, among the single‐stranded oligonucleotides, the lowest K_D_ value (2.7 nm) was observed for a G‐rich oligonucleotide (5′‐GGGUCUGUGGGGUC‐3′, henceforth called ‘G‐rich short RNA’) which suggested that FASTK might have a propensity for G‐rich stretches in single‐stranded RNAs. To further examine this hypothesis, we tested modifications of this G‐rich oligonucleotide where guanines were replaced with other three nucleotides (Fig. 2D). The results indeed emphasized the propensity of FASTK for binding G‐rich RNA stretches. *K*_*D*_ values for A‐rich (5′‐AAAUCUGUAAAAUC‐3′) and U‐rich (5′‐UUUUCUGUUUUUUC‐3′) short RNAs increased by one order of magnitude (78.4 nm and 63.2 nm, respectively), and two orders (212.3 nm) for C‐rich short RNA (5′‐CCCUCUGUCCCCUC‐3′).

### RNA binding by FASTK does not depend on G‐quadruplex formation

**Fig. 3:**
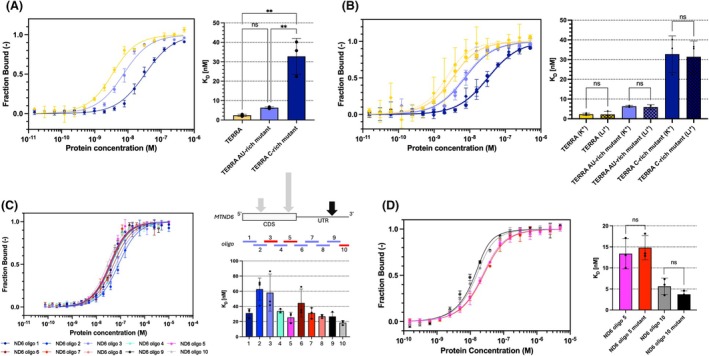
Microscale thermophoresis analysis of FASTK∆N1‐75 binding to guanine‐rich RNAs. (A) Binding of G‐quadruplex forming TERRA RNA and its mutants. (B) Binding of TERRA RNA and its mutants in the presence of potassium and lithium ions. (C) Binding of ten ND6 transcript fragments (oligo 1–10, red color indicates a predicted G4 structure) derived from the CDS and UTR of the shortest ND6 mRNA. The arrows represent the data previously obtained by Jourdain *et al*.: their location indicates the three main peaks in the number of reads in the RNA‐IP experiment (with the highest peak located in the region of oligo 5), and the dark color of the arrow corresponds to the identified protection site [2]. (D) Binding of RNA oligonucleotides 5 and 10 of ND6 transcript, compared to their binding after the introduction of G4‐killing mutations. The binding curves were plotted as a bound fraction of RNA against a protein concentration. Each measurement point was derived from three technical replicates and error bars represent the SD. Statistical significance of pairwise comparisons was calculated with Tukey's multiple comparisons tests for an ordinary one‐way analysis of variance (ANOVA): *P* > 0.05 (ns); *P* ≤ 0.01 (**).

Our results were consistent with previous reports that FASTK binds the guanine‐rich ND6 mRNA and its precursors, which are transcribed from the light strand of the mitochondrial genome [2]. ND6 mRNA is theoretically prone to form G‐quadruplex structures (G4s). We tested FASTK for its ability to bind a model RNA G‐quadruplex, derived from the telomeric repeat–containing RNA, TERRA (5′‐UUAGGGUUAGGGUUAGGGUUAGGG‐3′) [31, 32]. In order to achieve proper folding of TERRA into its G4 secondary structure, the oligonucleotide was subjected to slow thermal annealing in the presence of potassium ions. The binding assay revealed that FASTK indeed binds the TERRA G4 structure with a K_D_ comparable to the one obtained for G‐rich short RNA (2.4 nm) (Fig. 3A). Along with TERRA, we tested two mutants designed to disrupt the formation of the G‐quadruplex – the first with point mutations replacing central guanines in guanine triads with adenines and uracils (TERRA AU‐rich mutant), and the second with all guanines substituted with cytosines (TERRA C‐rich mutant) (Fig. 3A). No significant difference in binding was observed for the AU‐rich mutant – *K*_*D*_ increased less than three‐fold (6.4 nm). However, a more pronounced difference in binding resulted from replacing all guanines with cytosines – *K*_*D*_ in this case was one order of magnitude higher than for TERRA (32.8 nm). Similarly, the gel shift assay showed a preference towards G‐abundant TERRA RNA and visibly lower affinity towards AU‐ and C‐rich TERRA mutant (Fig. S4). The FASTK‐RNA complex did not enter the gel, which may be due to the formation of large protein‐RNA assemblies, possibly with multiple copies of FASTK per RNA molecule. However, other reasons, such as the high pI value of 9.5 for FASTK∆N1‐75, or possibly an extended shape of the complex, may also have contributed to the retention of protein‐RNA complexes in the wells. Nonetheless, free RNA could be quantified for estimation of the apparent *K*_*D*_ values, and these correlated well with MST data (though their values were generally higher). To further examine if FASTK is capable of binding RNA G‐quadruplexes, the same set of TERRA oligonucleotides was subjected to an annealing reaction in a regular annealing buffer (50 mm Tris/HCl, 50 mm KCl, 50 mm NaCl; pH 7.5) and a G4‐disfavoring buffer containing 50 mm LiCl instead of KCl, to avert the formation of guanine tetrads. Comparison of binding in the presence of K^+^ or Li^+^ proved that the secondary structure of tested oligonucleotides did not have an impact on interactions with FASTK. Regardless of the annealing buffer composition, the *K*_*D*_ values remained unchanged (Fig. 3B). We also compared FASTK binding to TERRA in the absence of Na+ in the RNA annealing and binding buffers (Fig. S5A), to exclude interference of Na+ with G4 stability (this cation is expected to have an intermediate stabilizing effect on G4s). FASTK showed better affinity in the presence of K+ than in Li+, both for TERRA and its mutants. We could not conclude that the relatively improved affinity in the presence of K+ can be attributed to the FASTK preference for G4s, as Li+ might have a slightly adverse effect on FASTK in general. Moreover, in the absence of Na+, the TERRA C‐rich mutant showed improved binding over the AU‐rich mutant, which was in contrast to the trend observed previously both in MST and EMSA. Therefore, our optimized binding conditions for FASTK retained both Na+ and K+, and the introduction of G4‐disrupting mutations in the RNA remained the most suitable method of testing the RNA binding preference of FASTK.

Consistent results were obtained when we tested the binding of ten ~125 nt long oligonucleotides derived from the mitochondrial ND6 mRNA (Fig. 3C). Interestingly, two of the tested oligonucleotides, which exhibited the lowest K_D_ values in FASTK binding assays (oligo 5–25.5 nm, oligo 10–18.5 nm) comprised G‐rich sequences capable of forming G4s structures. In order to assess the relevance of these structures in FASTK–RNA interactions, oligonucleotides were modified to disrupt the formation of putative G‐quadruplexes by introduction of adenines and uracils between the guanine nucleotides (Table S2). Such mutations had no impact on interactions with FASTK (Fig. 3D), which might suggest that FASTK binds G‐rich stretches of single‐stranded RNAs that might be separated with adenines and uracils, and which do not necessarily have to constitute a G‐quadruplex structure.

The influence of the G4‐disrupting mutations on the secondary structure of all the tested oligonucleotides was confirmed by the detection of G‐quadruplexes with the Thioflavin T dye (Fig. S5B). The intensities of the fluorescence emission spectra measured for TERRA and ND6 oligonucleotides mutants were visibly weaker than for the original RNAs, suggesting that the introduced mutations indeed decreased the formation of G‐quadruplexes. We attempted to characterize the complex of FASTK with the model TERRA RNA by SEC‐MALS analysis. Based on the SEC‐MALS data (Fig. 1E), we concluded that FASTK exists as a monomer in the solution, but we could not exclude the possibility of the formation of higher‐order oligomeric states upon binding to RNA, nor the binding of more than one TERRA molecule to a single FASTK molecule. However, when FASTK∆N1‐75 was mixed at a 1:1 molar ratio with TERRA RNA, the resulting complex was insoluble and could not be observed by SEC‐MALS (Fig. S5C). Regardless of whether the protein was mixed with RNA at a high concentration or first mixed at a low concentration and then concentrated as a complex – this mixture had a tendency to precipitate from the solution. After centrifugation of such complex, only a tiny peak was observed by SEC‐MALS for the FASTK monomer, suggesting that FASTK forms higher‐order protein‐RNA assemblies with a propensity to aggregate upon binding to RNA.

### FASTK protects bound RNA from degradation by the degradosome

**Fig. 4:**
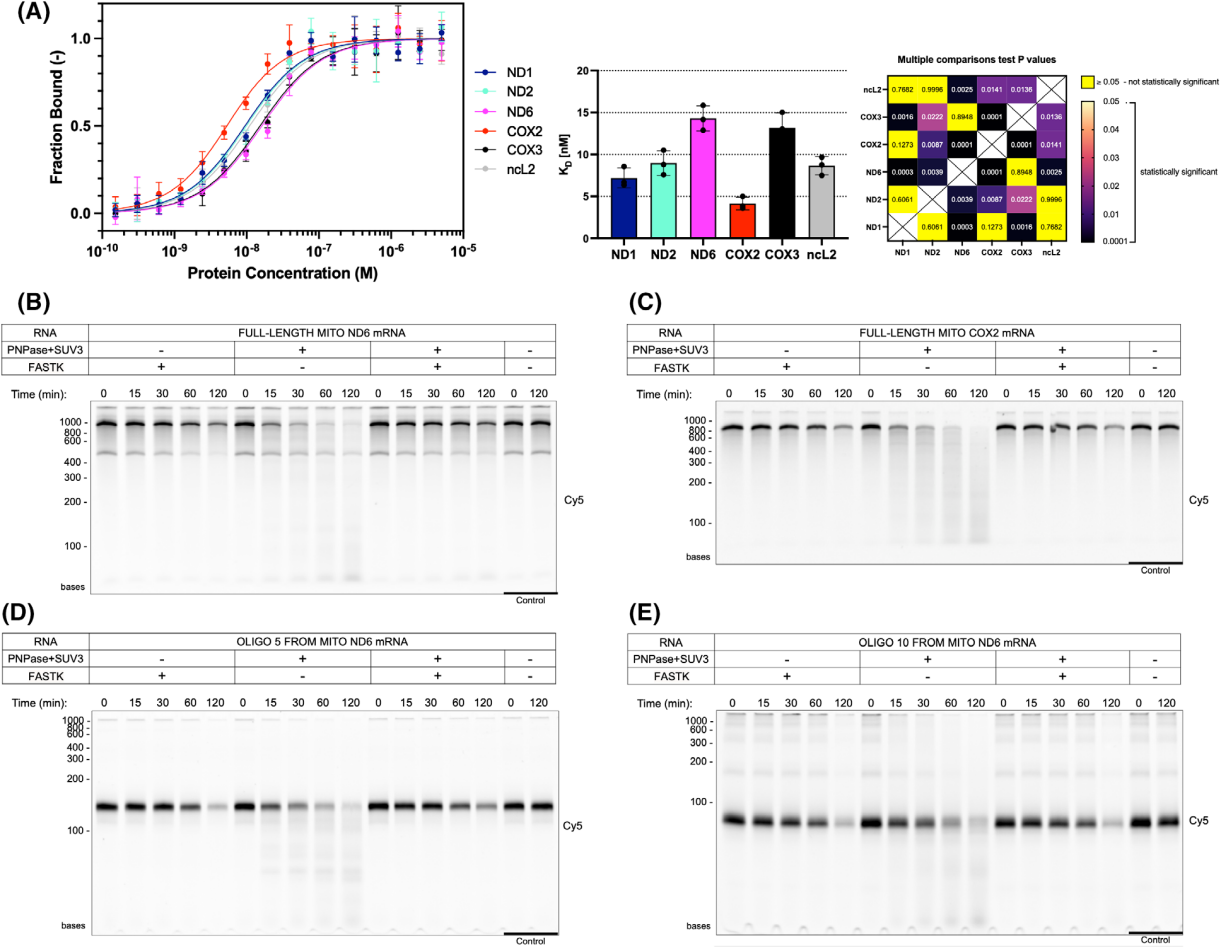
Binding and protection of mitochondrial transcripts by FASTK∆N1‐75. (A) Binding of six mitochondrial transcripts by FASTK∆N1‐75 protein, determined by microscale thermophoresis. The binding curves were plotted as a bound fraction of RNA against a protein concentration. Each measurement point was derived from three technical replicates and error bars represent the SD. Mean K_D_ values of individual experiments with SD are shown. The *P*‐values of Tukey's multiple comparisons tests for ANOVA comparing mean *K*_*D*_ values are presented as a heatmap. A significant result is *P* < 0.05. X indicates no valid comparison. *In vitro* degradation of Cy5‐body‐labeled ND6 mRNA (B), COX2 mRNA (C), and ND6 mRNA fragments (oligo 5 – D, oligo 10 – E) by recombinant PNPase and Suv3p proteins and the impact of FASTK on the RNA degradation. Reaction samples were collected at 0, 15, 30, 60, 120 min time points. The two last lanes present RNA after incubation without the addition of recombinant proteins. Visualized is the Cy5 signal after denaturing PAGE (*n* = 1). The experiments in B–E were performed once due to the limited availability of SUV3/PNPase and the time constraints, since to ensure similar activity of proteins, these degradation experiments were performed alongside each other. The qualitative assessment of degradation efficiency should, however, be performed across conditions within a single experiment, in comparison to controls visualized on the same gel.

We next tested FASTK binding to several full‐length, *in vitro* transcribed, mitochondrial mRNAs (Fig. S6A). Surprisingly, all these transcripts bound very well regardless of their calculated G % or predicted G4 content (Table S3), with COX2 mRNA having the strongest binding affinity (Fig. 4A). We selected ND6 and COX2 mRNAs for degradation assays to investigate the effect of FASTK binding on the downstream functional consequences.

Previously, cell‐line studies using RNA‐IP revealed that FASTK binds ND6 mRNA and its precursors and cooperates with the mitochondrial degradosome to generate mature ND6 by preventing its CDS and 3′ UTR degradation [2]. To test the role of *in vitro* FASTK activity in RNA protection, we set up an RNA protection assay containing bacterially expressed and purified components of the mitochondrial degradosome complex (the 3′‐5′ exoribonuclease polynucleotide phosphorylase PNPase and the helicase Suv3p), FASTK∆N1‐75, and *in vitro* transcribed Cy5‐labeled mitochondrial mRNAs. The outcome of our RNA protection assays confirmed the observations made formerly in cells that FASTK inhibits degradosome activity. All tested RNA substrates were degraded by the degradosome; however, in the presence of FASTK, the degradation activity was notably diminished (Fig. 4B–E). In accordance with the RNA binding preference studies carried on full‐length mitochondrial transcripts, the protection activity seemed to be independent of the nucleotide composition of the used substrates. Both the G‐rich ND6 and C‐rich COX2 mRNAs were equally protected in reactions containing recombinant FASTK. This protective effect also extended to the shorter ND6 oligonucleotides 5 and 10. We observed some decrease in the concentration of the full‐length transcripts with the addition of FASTK to the reactions with prolonged incubation times, and we investigated the possibility that this effect might be attributed to residual FASTK nuclease activity or a nuclease contamination. However, this decrease in full‐length RNAs was not associated with any intermediate degradation products derived from the body‐labeled substrates, unlike the clear ladder visible in all degradosome‐treated samples. Mass spectrometry analysis of two independently produced batches of our recombinant FASTK did not indicate any abundant nuclease contaminants (Table S4). Moreover, we have already observed solubility problems with FASTK‐RNA mixtures. In line with these observations, we thus speculated that the most likely cause for RNA loss might be that RNA‐FASTK complexes precipitate during the prolonged incubation of reaction mixtures.

We addressed the lack of discrimination by FASTK against the sequence of longer RNAs by trying to pinpoint the exact regions bound by FASTK. We performed a footprinting assay using ND6 oligo 5, a suitably sized transcript for this analysis (Fig. S6B). Although FASTK had a general protective effect, we were not able to identify any specific region strongly protected by FASTK, which suggests that FASTK was bound all along the transcript. This is in line with the poor sequence specificity of FASTK as shown by SELEX, and its proposed role as a protective, physical barrier from the access and activity of nucleases.

### A low‐resolution structural model of FASTK reveals a potential RNA‐binding interface

**Fig. 5:**
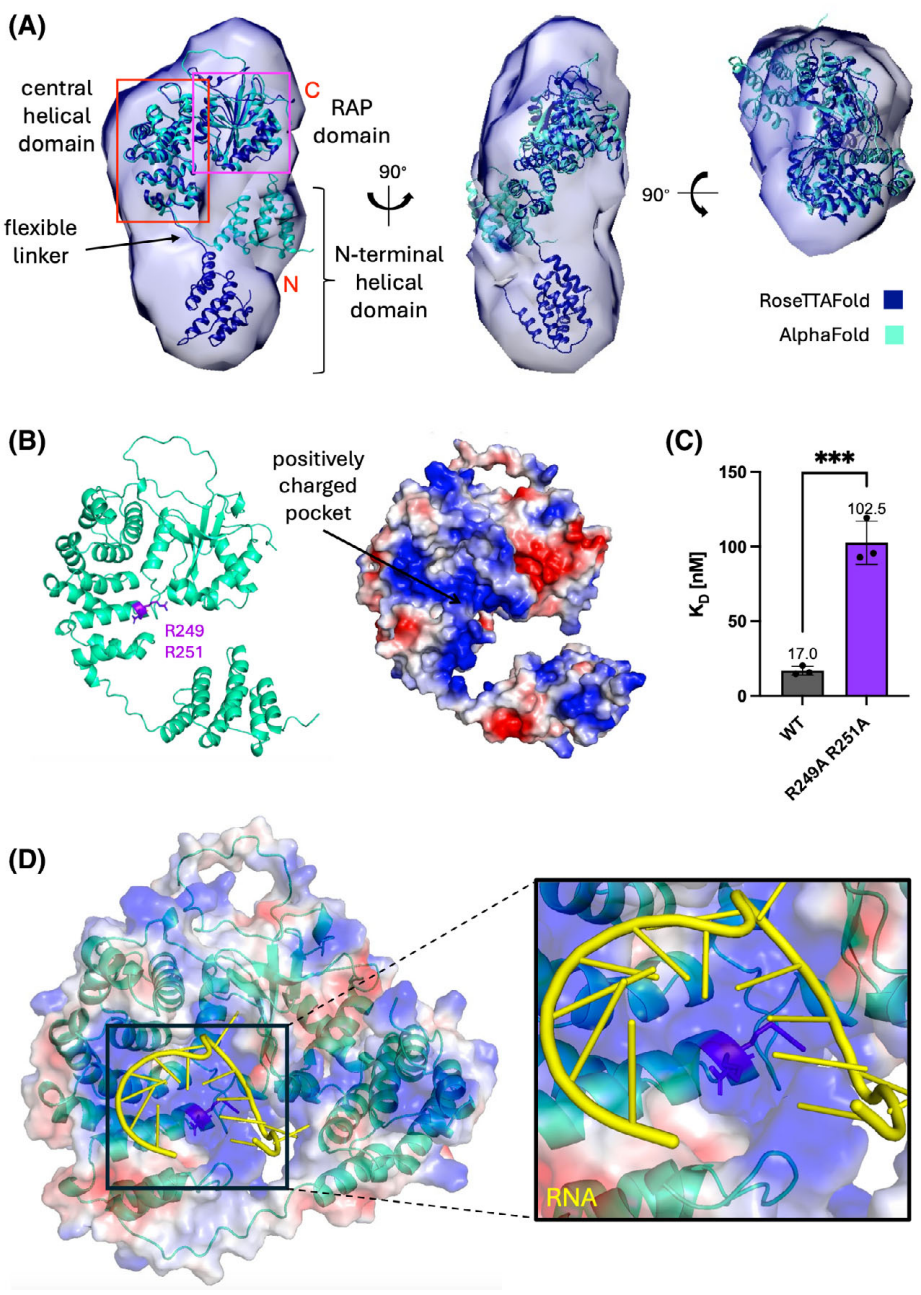
Analysis of FASTK∆N1‐75 structure and RNA‐binding disrupting mutations. (A) Front, side, and top views of FASTK∆N1‐75 prediction models fitted in the electron density generated by DENSS algorithm [33] based on the SEC‐SAXS measurements. Model created with RoseTTAFold [34] (dark blue) was fitted into the map using the Fit in Map tool in UCSF Chimera software, then the AlphaFold2 [35] model (aquamarine) was aligned to it using the MatchMaker comparison tool. (B) Map of protein contact potential generated for AlphaFold2 prediction model of FASTK∆N1‐75 allowed discovering positively charged pocket, putatively involved in RNA binding. (C) Microscale thermophoresis analysis confirmed that mutations in residues R249 and R251 significantly lowered FASTK∆N1‐75 ability to bind fragment 5 of mitochondrial ND6 RNA. Mean *K*_*D*_ values (indicated above the bars) were derived from three technical replicates, and error bars represent the SD. Statistical significance of pairwise comparisons was calculated with Tukey's multiple comparisons tests for an ordinary one‐way analysis of variance (ANOVA): *P* ≤ 0.001 (***). (D) The AlphaFold3 [36] prediction of FASTK∆N1‐75‐RNA complex structure utilizes RNA binding site encompassing residues R249 and R251.

The optimized expression and purification procedure of two FASTK protein constructs resulted in excellent, homogenous samples suitable for structural studies. Although FASTK eluded crystallization, we obtained the first experimental structural information for FASTK from SEC‐SAXS measurements (Table S5). We calculated the electron density distribution and compared the reconstructed structural envelopes [33] for FASTK∆N1‐75 (Fig. 5) and FASTK∆N1‐168 (Fig. S7) with structural predictions generated by various machine learning methods. To validate our low‐resolution experimental densities, we fitted FASTK atomic models created with RoseTTAFold [34] or AlphaFold2 [35]. The atomic models fit the experimental data well in the C‐terminal region of the protein, comprising the conserved FASTKD protein family domains ‐ FAST_1, FAST_2 and RAP. The alignment of the two models suggested that a flexible proline‐rich linker (amino acids 188 to 196) might allow the protein to adopt both an open and a closed conformation. While the RoseTTAFold model displayed an open conformation with the N‐terminal helical domain distant from the C‐terminal RAP domain, the AlphaFold2 model presented a more compact conformation with the helical domain angled towards the C‐terminal region (Fig. 5A). The AlphaFold3 model of FASTK∆N1‐75 assumed an even more closed conformation of the N‐terminal helical domain, which further suggests its possibly mobile nature.

The analysis of the surface electrostatic potential of FASTK∆N1‐75 models revealed a positively charged region within the central helical domain of the protein—a putative RNA‐binding site (Fig. 5B). To probe the localization of the putative RNA‐binding interface and the accuracy of the predicted model, we mutated several batches of positively charged residues and characterized RNA binding by the mutant FASTK∆N1‐75. Residues embedded within the central pocket (R249, R251, K275, K279, and R286), alongside other positively charged, exposed amino acids (Fig. S8A) were changed to alanines or methionines to neutralize their charges. We purified mutants and evaluated their binding of the ND6 oligo 5. Mutations at residues R249 and R251 decreased RNA binding 5‐fold when compared to the wild‐type protein (Fig. 5C), and mutations at the nearby K275, K279, and R286 were also markedly effective (Fig. S8B,C), whereas other mutants had no direct effects (and in two cases caused reduced protein solubility). Finally, AlphaFold3 [36] which allows facile prediction of protein‐RNA complexes, also placed G‐rich short RNA in the vicinity of the R249‐R251 region (Fig. 5D).

Comparison with the crystal structure of FASTKD4 [16] revealed that the same positively charged site is conserved in other paralogs, with the key FASTKD4 residues K337, K343, R378, and R581 located in the vicinity of, or being equivalent to, FASTK R249, R251, and R286 (Fig. S9). FASTKD4 K453 corresponds to FASTK Y357, preceded by R356. Although the two RNA‐binding interfaces seem similar, they must still contain differences responsible for the different substrate preference, with FASTKD4 protecting poly(A) tails of mRNA.

## Discussion

Direct RNA binding by human FASTK has been suggested by evidence from human cell line studies and backed by the involvement of its homologs in RNA metabolism in other species. Here, we provided the first direct evidence of RNA binding by FASTK, due to the availability of highly purified and homogenous samples of the recombinant protein. We characterized the RNA binding preference of FASTK in terms of sequence specificity, RNA structure, and nucleotide content. Our *in vitro* binding and degradation assays showed that purified FASTK is able and sufficient to recapitulate previous *in vivo* reports, at least regarding efficient binding to the central ND6 mRNA region and the role of a protective barrier against RNases.

Our success in FASTK purification might be partially due to the careful removal of the bound (bacterial) RNA, since we observed decreased solubility of FASTK‐RNA mixtures. Purified FASTK was monomeric, but in our hands precipitated upon the addition of RNA, which suggests formation of higher‐order protein‐RNA assemblies (condensates), reminiscent of a phase transition. This is in line with the descriptions of FASTK as a component of RNA granules both in the mitochondria and in the cytoplasm [2]. The likely flexible nature of FASTK has so far rendered it poorly suitable for high‐resolution studies, but our low‐resolution SAXS data agree with most of the current machine learning structural predictions. The SAXS data suggest an extended, open conformation of FASTK in solution, whereas the closed conformation predominated in AlphaFold predictions – the experimental data overcome a known caveat of the current state of the machine learning methods [34, 35, 36] which cannot fully recapitulate protein dynamics such as the positioning of the mobile parts of the protein. Our mutagenesis study provided evidence for the localization of the putative RNA‐binding interface in the central region of FASTK; it is interesting to speculate that FASTK might ‘clamp’ around RNA molecules using the R249/R251‐lined ‘saddle’ and the flexibly connected N‐terminal domain.

We were able to delve into the RNA‐binding properties of FASTK using the most amenable short oligonucleotides. We failed to detect any RNA sequence‐specific features, but FASTK was sensitive to the nucleotide content of RNA, with a preference towards G‐rich oligonucleotides and discrimination against C‐rich ones. This propensity for G‐rich oligonucleotides was affected by the strand pairing, with ssRNA being preferred over dsRNA or stem loops. FASTK binding to RNA was relatively insensitive to the introduction of single adenine or uracil nucleotides in the G‐tracts, as well as to the formation of G‐quadruplexes. Overall, our results suggest that the formation of G4s is tolerated and might be even beneficial, but is not required for FASTK binding. This indicates that FASTK has permissive and robust RNA binding properties. Unexpectedly, in a small screen of six full‐length mitochondrial mRNAs, all were bound well by FASTK regardless of their G‐ or G4‐content. This suggested that longer transcripts might perhaps benefit from cooperativity or a local concentration effect to promote the coating of the RNA by FASTK. This coating along the body of RNA was also suggested by the uniform protection of ND6 oligo 5 in a footprinting assay. Notably, FASTK binding to COX2 mRNA was tighter than to the ND6 mRNA, the best‐described natural target of FASTK. Possibly, FASTK alone is able to discriminate nucleotide content only for short oligonucleotides or isolated sites, whereas for longer transcripts, the specificity is lost due to overall robust RNA binding. This might be especially true if cooperative coating of the RNA takes place upon crossing a certain initial binding threshold or a local concentration. Regulated access to preferred ‘initial’ sites on RNA might be the deciding factor for FASTK specificity. *In vitro* folded, isolated mRNAs most likely differ from their physiologically relevant structures and conformations found *in vivo*, and their accessibility in the cell is expected to be further regulated by proteins, cellular machineries, membranes, and localization. Similarly, in the case of FASTK binding to RNA, the preferences of this protein demonstrated here using shorter oligonucleotides will be compounded by many additional factors in the cell, especially those regulating RNA accessibility or FASTK localization. While the intrinsic properties of FASTK might direct it to binding G‐rich transcripts such as ND6, we expect other factors to increase the selectivity of FASTK *in vivo*.

**Fig. 6:**
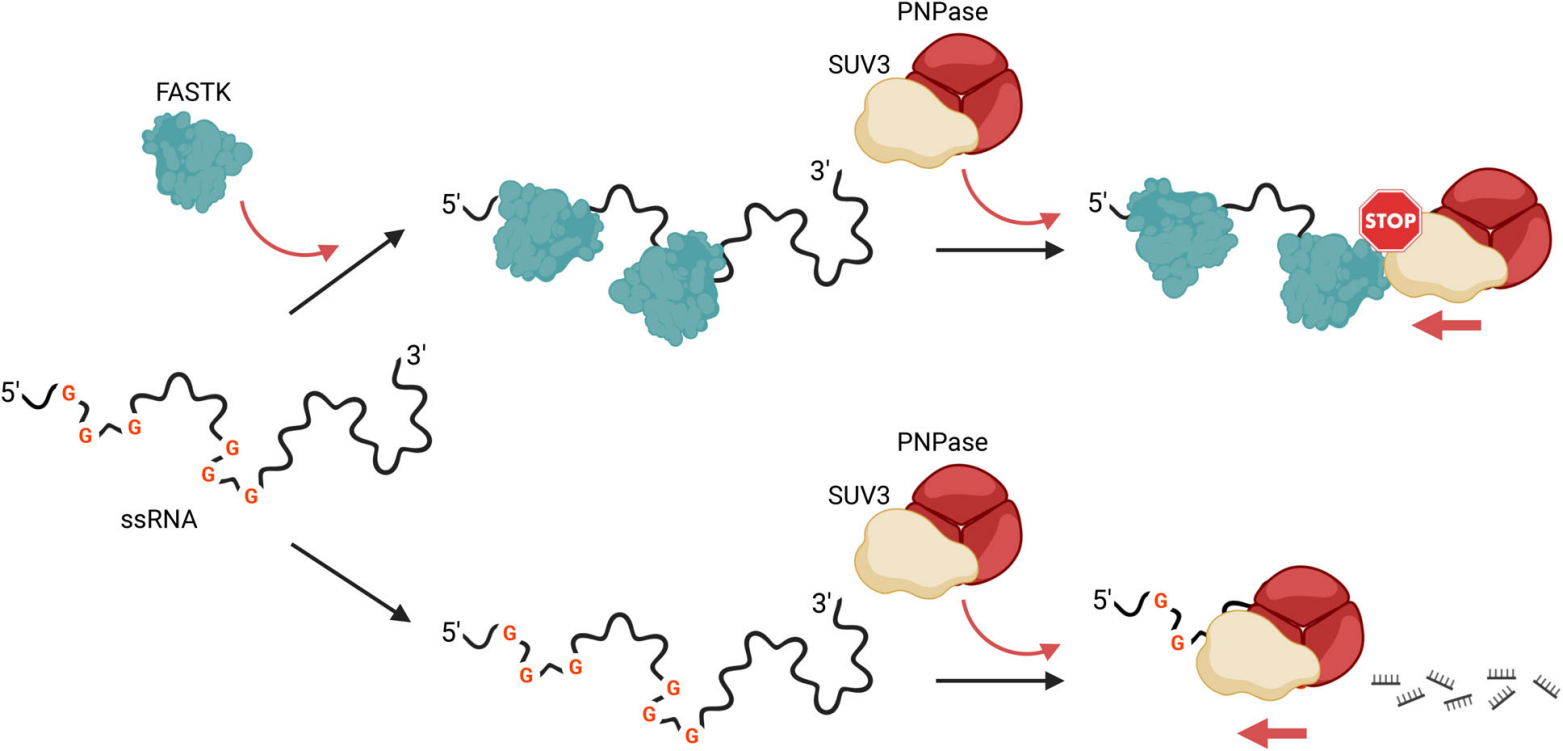
FASTK binds G‐rich sites of single‐stranded mRNA. By protecting these regions, FASTK inhibits the processing of the mitochondrial degradosome complex (PNPase and SUV3), thereby preventing exonucleolytic decay. Created in BioRender. Gorna, M. (2025) https://BioRender.com/40ycqx7

We demonstrated that the addition of FASTK alone was sufficient to compete with and inhibit degradation by the degradosome, which recapitulates well the known protective effect of FASTK on ND6 mRNA. This mechanism seems to correlate with direct RNA binding by FASTK, as it was also observed with other substrates. FASTK binding selectivity might thus drive the protective effect of FASTK. In the simplest approximation, RNA binding by FASTK may result in a physical obstacle due to FASTK‐RNA interactions outcompeting degradosome processivity (*i.e*., the degradosome is deterred since it is unable to displace FASTK ‘markers’ deposited on G‐rich sites or G‐quadruplexes (Fig. 6)). The results of the footprinting assay also suggest that FASTK forms a general protective barrier from other nucleases (RNase T1) and factors (lead). Our observed best affinity for ND6 oligo 5 coincides with the location of the highest peak of reads in this region reported in RNA‐IP experiments by Jourdain *et al* [2]. This site might indeed be the preferred binding site both *in vitro* and in cells. However, even in the same study, the reported protection site did not correspond to this peak, but was shifted towards a minor peak nearer the 3′ end of the transcript (region at the start ND6 oligo 9). If protection occurs further from the preferred binding site, this might go hand in hand with our proposed mechanism in which FASTK, after initial binding and ‘nucleation’ might coat the RNA to extend a physical barrier‐like protection from nucleases along its body. Alternatively, liquid–liquid phase separation might be yet another mechanism through which FASTK may regulate RNA localization and accessibility *in vivo*. These downstream effects are not easily disambiguated *in vitro* nor *in vivo*, as single FASTK‐RNA complexes are difficult to obtain for study, but future single‐molecule studies in cells could help with this issue.

Our observations explain how FASTK might regulate the degradation of ND6 and other G‐rich transcript fragments in the mitochondria, but the question arises how the same binding properties of FASTK might be used in other cellular compartments which lack the degradosome. In the nucleus, FASTK might bind G‐rich sequences in precursor or mature mRNAs and regulate their splicing or stability through interplay with other proteins. Although locally G‐enriched regions in transcripts are ubiquitous and therefore their comprehensive analysis may be difficult, specific G4‐forming regions have started to be characterized for their role in RNA stability and accessibility [37]. G4s are highly enriched at splice site regions and are preferably formed within the non‐template strand of the genome, leading to their formation on the pre‐mRNA [38, 39, 40, 41, 42]. Moreover, G4s are proposed to play a role in stress granule formation via their interactions with RBPs [43, 44, 45, 46], which correlates with the reports of FASTK as a scaffolding protein mediating transitions between stress granules and processing bodies [47]. Indeed, mRNA known to be regulated by FASTK contains sequences predicted to form G4s— for example, the 5′UTRs of cIAP [48] and Bcl‐2 mRNAs [49]— and these G‐rich regions might serve to recruit FASTK. Our results suggest that the role of G‐rich RNA recognition by FASTK and its role in the nuclear RNA metabolism and cytoplasmic RNA granules should be the subject of future studies.

## Materials and methods

### Cloning of plasmids for bacterial expression

The full list of vectors and DNA starters used in the study is provided in the Supplementary Tables S6 and S7. The expression vector pCA528, containing a sequence of His6‐SUMO tag, was a kind gift from Prof. Owen Pornillos (University of Virginia). The vector used for the expression of Suv3p protein was described previously [20]. The pGEX‐4 T1 vector used for cloning of GST‐FASTK∆N1‐168 for the SELEX assay was a kind gift from Dr. Martyna Nowacka (University of Warsaw). Vectors pDONR223‐FASTK and pET28a_TIA1 were purchased from Addgene (#23712 and #106095).

All constructs for the bacterial expression of proteins (pCA528‐FASTK∆N1‐75, pCA528‐FASTK∆N1‐168, pCA528‐TIA1‐RRM23 and pGEX‐GST‐FASTK∆N1‐168) were obtained by SLIC cloning [50, 51]. FASTK encoding DNA was amplified from the pDONR223‐FASTK plasmid and was cloned into the pCA528 vector, followed by transformation to *E. coli* Top10 and kanamycin selection. The mutagenesis of pCA528‐FASTK∆N1‐75 was performed by PCR amplification of whole plasmid DNA using a suitable pair of primers introducing intended mutations (Table S7). Amplification was followed by plasmid circularization by simultaneous phosphorylation and ligation of plasmid ends, and transformation of the *E. coli* Top10 and kanamycin selection. For the cloning of pCA528‐TIA1‐RRM23, DNA of TIA‐1 protein (aa 80–292) was amplified from the pET28a‐TIA1 plasmid and was cloned into the pCA528 vector, followed by transformation to *E. coli* Top10 and kanamycin selection. For the cloning of pGEX‐GST‐FASTK∆N1‐168, DNA of FASTK ORF was amplified from the pDONR223‐FASTK plasmid and was cloned into the pGEX‐4T1 vector, followed by transformation to *E. coli* Top10 and ampicillin selection.

### Cloning and *in vitro* transcription of mitochondrial mRNA

All RNA oligonucleotides up to 25 nucleotides long were purchased from either Merck or Future Synthesis. The pCR™‐Blunt II‐TOPO™ vector was a kind gift from Dr. Marcin Ziemniak (University of Warsaw). The mitochondrial DNA isolated from HeLa cells was a kind gift from Dr. hab. Roman Szczęsny (Institute of Biochemistry and Biophysics, Polish Academy of Sciences). Human mitochondrial *MT‐ND6* gene was amplified from mitochondrial DNA and inserted into pCR™‐Blunt II‐TOPO™ vector for further *in vitro* transcription. The sequence of the *MT‐ND6* gene was amplified by PCR reaction and used in the TOPO™ cloning reaction according to the manufacturer's instructions. For *in vitro* transcription of fragments of ND6 mRNA bearing mutations removing possible formation of G‐quadruplexes, the pCR™‐Blunt II‐TOPO™‐ND6 plasmid was mutated using a procedure analogous to the one described above.

pCR™‐Blunt II‐TOPO™‐ND6 vector was used as a template for *in vitro* transcription of full‐length ND6 mitochondrial transcript, its ~100 nt long fragments, and mutants. The selected DNA sequences were amplified by PCR reaction with forward starters containing overhangs with the sequence of T7 RNA polymerase promoter (5′‐TAATACGACTCACTATAGG‐3′). The list of starters used in the experiment is specified in Table S8. 20 μL *in vitro* transcription reactions were prepared on ice with the following ingredients: DNA template (300–1000 ng), 1× T7 RNA polymerase buffer, rNTPs (5 mm each*), 1 mm DTT, 3% DMSO, 0.25 μL RiboLock RNAse inhibitor (Thermo Fisher Scientific, Waltham, MA, USA), 2 μL home‐made T7 RNA polymerase. * For transcription reaction of fluorescently labeled RNAs, 0.5 mm Cy5‐CTP or Cy5‐UTP was included in the reaction, and the concentration of the corresponding unlabeled ribonucleotide was lowered to 3.5 mm. Reactions were incubated at 37 °C for 4 h and transferred on ice. The template DNA was then digested by addition of 1 U of TURBO DNAse (Thermo Fisher Scientific) and incubation at 37 °C for another 30 min. The reactions were quenched by addition of 50 mm EDTA. The products of the transcription were then purified with QIAGEN RNeasy Mini Kit according to the manufacturer's instructions and visualized by RNA PAGE.

*In vitro* transcriptions of additional mitochondrial transcripts (*ncL2*, *COX2*, *COX3*, *ND1* and *ND2*) were performed using pRS147X vectors, which were a kind gift from dr hab. Roman Szczęsny. pRS147X vectors have a built‐in sequence of T7 RNA polymerase promoter, a sequence of each transcript and restriction enzymes sites, enabling adjustment of the length of transcript's poly(A) tail. The *in vitro* transcription reactions were prepared as described above and products were analyzed by RNA PAGE.

### RNA annealing

The RNA annealing procedure was performed in 1× Annealing Buffer (50 mm Tris/HCl pH 7.5, 50 mm KCl, 50 mm NaCl) with the use of a thermal cycler and according to the following steps: (i) 5 min denaturation at 95 °C, (ii) slow cooling to RNA's T_M_, (iii) 30 min incubation at RNA's T_M_, and (iv) slow cooling to 4 °C. The whole procedure was spread over time to take 2 h. RNAs were then transferred on ice and immediately used for further experiments.

### Thioflavin T assay

The Thioflavin T reactions were prepared by mixing 2 μm Thioflavin T (Thermo Fisher Scientific), 2 μm annealed RNA in 1× Annealing Buffer. Reactions were then transferred to 96‐well plates (PS, F‐bottom/chimney well) (Greiner) and measured by Tecan M200 Pro Plate Reader with excitation at 430 nm and the fluorescence emission scan between 460 and 700 nm every 2 nm.

### Protein expression

The overexpression of FASTK∆N1‐75, FASTK∆N1‐168 (Table S9), GST‐FASTK∆N1‐168, TIA1‐RRM23, PNPase, and Suv3p proteins was performed in *E. coli* BL21‐CodonPlus(DE3)‐RIL cells with the induction of protein expression at OD_600_ of 0.5–0.8 with 1 mm IPTG, followed by overnight incubation at 20 °C.

### Purification of recombinant FASTK proteins

Harvested cell pellets were suspended in Lysis Buffer (150 mm Tris/HCl pH 8.5, 5% (v/v) glycerol, 500 mm NaCl, 200 mm ammonium sulfate, 10 mm arginine, 10 mm glutamic acid, 5 mm β‐Me), freshly supplemented with 1 mm PMSF, 10 mm MgCl_2_, 1 mg·mL^−1^ hen egg lysozyme, and 10 μg·mL^−1^ DNase I, and incubated for 1 h at 4 °C on a rocking platform. Cells were disrupted by sonication (7 min, 2 s on/2 s off, 60% amplitude) on ice. The lysate was centrifuged at 20 000 ***g***, 4 °C for 30 min, and the obtained supernatant was subjected to polyethyleneimine (PEI) precipitation of nucleic acid contaminations as described in [52]: the lysate supernatant was placed in a glass beaker on a magnetic stirrer at 4 °C, and 9% PEI solution was added dropwise to the supernatant until a 0.1% concentration was reached. The solution was left with stirring for 20 min and then centrifuged at 20 000 ***g***, 4 °C for 20 min.

The supernatant was then filtered through a 40 μm syringe filter and applied on HisTrap HP column (Cytiva, Marlborough, MA, USA) attached to a Bio‐Rad FPLC system (Bio‐Rad, Hercules, CA, USA) and equilibrated with HisTrap Buffer A (50 mm Tris/HCl pH 8.5, 5% (v/v) glycerol, 50 mm NaCl, 50 mm ammonium sulfate, 10 mm arginine, 10 mm glutamic acid, 5 mm β‐Me). The recombinant His6‐SUMO tagged protein was then eluted with an increasing gradient of HisTrap Buffer B (1 m Imidazole, 50 mm Tris/HCl pH 8.5, 5% (v/v) glycerol, 50 mm NaCl, 50 mm ammonium sulfate, 10 mm arginine, 10 mm glutamic acid, 5 mm β‐Me). Fractions containing the protein of interest were combined and dialyzed overnight at 4 °C with 50 mL of home‐made recombinant SUMO protease (1 mg·mL^−1^), against Dialysis Buffer (50 mm Tris/HCl pH 8.0, 5% (v/v) glycerol, 50 mm NaCl, 150 mm ammonium sulfate, 10 mm arginine, 10 mm glutamic acid, 10 mm EDTA, 10 mm imidazole, 5 mm β‐Me). The cleaved protein was then diluted 2× in HiTrap SP Buffer A (50 mm Tris/HCl pH 8.0, 5% (v/v) glycerol, 50 mm ammonium sulfate, 10 mm arginine, 10 mm glutamic acid, 5 mm β‐Me) to decrease the NaCl concentration and facilitate protein binding to the column. The solution was then applied on HiTrap SP column (Cytiva) and the recombinant protein was eluted with an increasing gradient of HiTrap SP Buffer B (50 mm Tris/HCl pH 8.0, 5% (v/v) glycerol, 1000 mm NaCl, 50 mm ammonium sulfate, 10 mm arginine, 10 mm glutamic acid, 5 mm β‐Me). Eluted protein fractions were then concentrated to a volume <4 mL using Amicon® Centrifugal Filter Units (10 kDa MWCO) by centrifugation at 3000–5000 ***g***, 4 °C for 10–15 min. Concentrated protein was centrifuged to remove all precipitates at 10 000 ***g***, 4 °C for 15 min and applied on HiLoad 16/600 Superdex 75 pg column (Cytiva), equilibrated in Storage Buffer (50 mm Tris/HCl pH 8.5, 5% (v/v) glycerol, 50 mm NaCl, 250 mm ammonium sulfate, 10 mm arginine, 10 mm glutamic acid, 1 mm DTT). The fractions containing clean protein were pooled, concentrated as described above, flash‐frozen in liquid nitrogen and kept at −80 °C until used. All significant samples obtained during expression and purification were analyzed by SDS/PAGE.

### Thermal shift assay

Purified FASTK∆N1‐75 at a concentration of 1 mg·mL^−1^, initially in buffer containing 50 mm Tris/HCl pH 7.5, 5% (v/v) glycerol, 400 mm NaCl, 10 mm arginine, 10 mm glutamic acid, and 0.5 mm TCEP, was mixed with 5× concentrated SYPRO™ Orange Protein Gel Stain (Invitrogen, ThermoFisher Scientific, Waltham, MA, USA) and 10× diluted components of Additive Screen (Hampton Research, Aliso Viejo, CA, USA) and Solubility Screen (Jena Bioscience, Jena, Germany) in a 25 μL reaction volume. Reactions were prepared on 384‐well PCR plates, sealed with an optically clear sheet of adhesive, briefly centrifuged, and subjected to a thermal gradient produced by a CFX384 Touch Real‐Time PCR Detection System (Bio‐Rad).

### Microscale thermophoresis

Purified FASTKΔN1‐75 protein was dialyzed against MST Buffer (1× PBS, 5% (v/v) glycerol, 1 mm DTT) at 4 °C for 2 h and centrifuged to remove all precipitates at 10 000 ***g***, 4 °C for 10 min. The concentration of protein was measured using a NanoDrop spectrophotometer and then protein was diluted in MST buffer to a concentration of 2 μm. Samples of annealed Cy5 labeled RNA oligonucleotides were diluted in MST buffer supplemented with 0.05% Tween‐20 to a concentration of 40 nm. Sixteen binding mixtures were prepared by 1:1 mixing of an RNA solution (constant concentration), with protein solution (a 2‐fold dilution series), resulting in final 20 nm RNA and up to 1 μm protein. For the MST measurements in the absence of sodium salt, the annealing and binding buffers contained either 100 mm KCl or 100 mm LiCl (instead of a mixture with NaCl), PBS was replaced with Tris/HCl pH 7.5, and FASTKΔN1‐75 was dialyzed into the binding buffer prior to MST assays. Here, the final concentrations in MST measurements were 8 nm RNA and up to 1 μm protein for TERRA, and 20 nm RNA and 4 μm protein for TERRA mutants. Reaction mixtures were pre‐incubated for 5 min on ice, centrifuged for 2 min, and loaded into capillaries. The measurements were performed using a Monolith NT.115 device (NanoTemper Technologies, Munich, Germany) at a constant temperature of 22 °C, according to the manufacturer's instructions. Each measurement point (mean ± SD) was derived from three technical replicates. The fitting of the dose–response curves to a one‐site binding model was performed using MO Affinity Analysis v.2.3 software, supplied by the device manufacturer. The fit curves were then exported and plotted as a bound fraction of RNA against a protein concentration using Prism 10 (GraphPad). The mean and SD of the *K*_*D*_ values obtained from three separate measurements were calculated and then the statistical significance of pairwise comparisons of *K*_*D*_ values was calculated using two methods: (i) ordinary one‐way analysis of variance (ANOVA) test with Tukey's multiple comparisons test or (ii) the two‐tailed unpaired parametric *t*‐test, where *P* > 0.05 (ns); *P* ≤ 0.05 (*); *P* ≤ 0.01 (**); *P* ≤ 0.001 (***); *P* ≤ 0.0001 (****).

### Electrophoretic mobility shift assay (EMSA)

Solutions of FASTKΔN1‐75 protein and RNA oligonucleotides were prepared as described above for MST measurements. The binding mixtures were prepared by mixing an RNA solution of constant concentration with a series of 2× dilutions of protein solution in the concentration range from 1000 to ~8 nm. Additionally, two control reactions were included—one containing protein alone at the highest concentration used, and the second control with RNA alone. Reactions were then incubated for 5 min on ice, mixed with 10× EMSA loading dye (30% (w/v) Ficoll 400 (Sigma‐Aldrich) in water, supplemented with Orange G dye (Leica)) and separated by native gel electrophoresis in TG buffer using 4–20% Mini‐PROTEAN® TGX Stain‐Free™ gels (Bio‐Rad), subjected to a 30 min pre‐run. Gels were resolved at 100 V until ready and visualized with a ChemiDoc Imaging System. The intensities of RNA bands were quantified using ImageLab software.

### Analysis of oligomeric state by SEC‐MALS

Purified FASTK∆N1‐75 protein was dialyzed against SEC‐MALS Buffer (1× PBS, 50 mm ammonium sulfate, 5% (v/v) glycerol, 1 mm DTT) for 2 h at 4 °C. The analysis was carried out by injecting 100 μL of purified protein at a concentration of 5 mg·mL^−1^ and onto a Superdex 200 Increase 10/300 GL column (Cytiva) equilibrated with SEC‐MALS Buffer and connected to NGC Scout 10 FPLC System (Bio‐Rad). A solution of bovine serum albumin in SEC‐MALS Buffer at the same concentration and volume was used as a calibration standard. Particle size and refractive index data were collected with a RefractoMax 520 Refractive Index Detector (ERC Inc) equipped with an in‐line miniDAWN TREOS static light scattering detector (Wyatt Technology, Santa Barbara, CA, USA). Data were recorded and processed using ASTRA software (Wyatt Technology) using a single dn/dc value of 0.185 mL/g [53].

### Analysis of recombinant FASTK∆N1‐75 by mass spectrometry

Proteins were precipitated by methanol/chloroform precipitation and suspended in 100 mm HEPES, pH 8.0, containing 5 mm TCEP and 10 mm CAA (2‐chloroacetamide). Samples were incubated with 0.4 μg trypsin overnight in a thermo‐shaker set to 37 °C. The digestion was terminated by adding trifluoroacetic acid (TFA) to a final concentration of 1% and digested peptides were desalted with a C18‐StageTip. Prior to LC–MS measurement, the peptide fractions were resuspended in 0.1% TFA, 2% acetonitrile in water. Chromatographic separation was performed on an Easy‐Spray Acclaim PepMap column (50 cm long × 75 μm, Thermo Fisher Scientific) at 55 °C by applying 60 min acetonitrile gradients in 0.1% aqueous formic acid at a flow rate of 300 nL/min. An UltiMate 3000 nano‐LC system was coupled to a Q Exactive HF‐X mass spectrometer (data‐dependent mode, resolution 30 000, m/z 200) via an easy‐spray source (all Thermo Fisher Scientific). Up to 15 of the most abundant isotope patterns with charges 2–5 from the survey scan were selected with an isolation window of 1.3 m/z and fragmented by higher‐energy collision dissociation (HCD) with normalized collision energies of 27, while the dynamic exclusion was set to 30 s. The maximum ion injection times for the survey scan and the MS/MS scans (acquired with a resolution of 15 000 at m/z 200) were 45 and 22 ms, respectively. The ion target value for MS was set to 3 × 10^6^ and for MS/MS to 10^5^, and the intensity threshold for MS/MS was set to ^4^2 × 10.

### Kinase activity assays

TIA1‐RRM23 protein purification procedure was performed as described in [54], with minor modifications. [γ^32^P]ATP (Hartmann Analytic), control protein kinase (SnRK2.6), and myelin basic protein (MBP) were a kind gift from Prof. Grażyna Dobrowolska (Institute of Biochemistry and Biophysics, Polish Academy of Sciences). The putative kinase activity of FASTK protein was analyzed *in vitro* in the presence of recombinant FASTK∆N1‐75 and recombinant TIA1‐RRM23 and [γ^32^P]ATP as substrates. The protein components of the reactions were combined in Kinase Assay Buffer (25 mm Tris/HCl pH 7.5, 5 mm EGTA, 1 mm DTT, 30 mm MgCl_2_). Reactions were then initiated by the addition of 50 mm ATP supplemented with 1 μCi of [γ^32^P]ATP and were immediately placed at 30 °C. After 40 min, the reactions were quenched by the addition of 5× SDS/PAGE Sample Loading Buffer (50 mm Tris/HCl pH 6.8, 2% (w/v) SDS, 10% (v/v) glycerol, 100 mm β‐Me, 0.01% Bromophenol Blue) and boiling for 5 min. 15 μL of each sample was separated by SDS/PAGE at 200 V, and phosphorylated substrates were visualized by autoradiography.

### Systematic evolution of ligands by exponential enrichment (SELEX)

The procedure was carried out according to the protocol described in [29]. GST‐FASTK∆N1‐168 protein was expressed in 100 mL of transformed BL21(DE3) RIL *E. coli* culture, induced with 1 mm IPTG, and cultured at 18 °C overnight. The harvested pellet was resuspended in 1× PBS supplemented with 1 mm PMSF, cells were lysed by sonication (3 min, 30s on/30s off, 40% amplitude) and centrifuged for 45 min at 17 000 ***g***, 4 °C. Centrifuged lysate was then mixed with 400 mL of Glutathione Sepharose beads (Cytiva) (washed with miliQ water and 1× PBS) and incubated for 1 h at 4 °C with slow rotation. Beads were then washed 3 times with 1× PBS, and protein was eluted in 2 portions (1 and 0.5 mL each) by incubation in GST Elution Buffer (50 mm Tris/HCl pH 8.5, 5% glycerol, 50 mm NaCl, 200 mm ammonium sulfate, 1 mm DTT, 10 mm reduced glutathione) with shaking at 1000 rpm, 4 °C for 15 min on a thermomixer. Eluted protein was decanted from the beads, flash‐frozen, and stored at −80 °C.

80 picomoles of purified GST‐FASTK∆N1‐168 were incubated for 1 h at 4 °C in 1 mL of SELEX Binding Buffer (20 mm Tris/HCl pH 7.5, 0.1% Triton X‐100, 100 mm NaCl, 1 mm DTT) with 30 mL of Glutathione Sepharose beads. After incubation, beads were washed, resuspended in 1 mL of SELEX Binding Buffer supplemented with 2 μg·mL^−1^ of poly dIdC (Sigma‐Aldrich) and mixed with 13 μg of *in vitro* transcribed library of random RNA oligonucleotides (Metabion). The binding reaction was incubated with rotation for 1 h at 4 °C. Beads were then washed with SELEX Washing Buffer (20 mm Tris/HCl pH 7.5, 0.1% Triton X‐100, 250 mm NaCl, 1 mm DTT), and bound RNA oligonucleotides were released by proteolysis of FASTK protein in 400 μL of SELEX Washing Buffer supplemented with 25 μg of Proteinase K (Invitrogen, ThermoFisher Scientific) for 1 h at 37 °C, 800 rpm with shaking. The eluted RNA was precipitated in 1 mL of ice‐cold 100% ethanol with the addition of 3 μL of linear acrylamide (Invitrogen, ThermoFisher Scientific) and stored overnight at −20 °C. The next day, RNA was pelleted by centrifugation at 4 °C, 15 000 rpm for 30 min, washed with cold 80% ethanol, and dried by incubation at 37 °C. The dried pellet was then resuspended in water and reverse‐transcribed with 1st Strand cDNA Synthesis Kit for RT‐PCR (AMV) (Roche) following the manufacturer's protocol. The obtained cDNA was then *in vitro* transcribed according to the procedure for T7 RNA polymerase and used in the subsequent selection reaction. After the second iteration of selection, the amplified cDNA was sequenced by Next Generation Sequencing with Genome Sequencer Illumina NovaSeq 6000 PE100 (the Genomics Core Facility, Centre of New Technologies, University of Warsaw). The obtained data were analyzed using the motif discovery tool MEME (Multiple Em for Motif Elicitation) [55, 56].

### RNA protection assays

The purification of Suv3p was performed according to the FASTK protein purification procedure. The only modification was introduced during the lysis of the pellets, when cells were homogenized using the PandaPLUS homogenizer. The recombinant human PNPase protein was prepared using a modified protocol for PNPase purification described in [57]. Degradation assay reactions were carried out at 37 °C in 50 μL reaction volume containing 50 or 200 nm RNA and indicated protein/s (FASTKΔN1‐75, Suv3p or PNPase) at concentrations of 1 μm each in RNA degradation buffer (20 mm Tris/HCl pH 7.5, 50 mm NaCl, 50 mm KCl, 1 mm MgCl_2_, 2 mm Sodium phosphate) freshly supplemented with 1 mm DTT, 1 mm ATP (Thermo Fisher Scientific) and 1 U·μL^−1^ RiboLock RNAse inhibitor (Thermo Fisher Scientific). Reactions were started by the addition of RNA previously subjected to slow annealing and placing at 37 °C. Full‐length mitochondrial RNA transcripts were used in the assay at the concentration of 50 nm, while shorter oligonucleotides (12–125 nt) were used at the concentration of 200 nm. At selected time points, 10 μL portions of reactions were mixed with 10 μL of 0.5 mg·mL^−1^ Proteinase K in Proteinase K buffer (100 mm Tris–HCl pH 7.5, 150 mm NaCl, 25 mm EDTA, 1% (w/v) SDS) and were immediately placed at 50 °C for 20 min. Samples were then removed from the thermo block, mixed with 10 μL of 2× formamide RNA loading buffer (95% (v/v) Formamide, 1 mm EDTA, 0.01% (w/v) Orange G dye (Leica), in deionized water) and flash frozen in liquid nitrogen. When all samples were collected, they were heated for 5 min at 95 °C, briefly centrifuged, and then the entire sample was applied and separated on a Urea PAGE gel in 1× TBE buffer. To enable good separation and visualization of short products of degradation, Urea PAGE was performed on 20 × 20 cm gels prepared using the UreaGel System from National Diagnostics. The gels were then stained by incubation with SYBR Gold Stain for 20 min in TBE buffer or were directly scanned with the Cy5 channel on ChemiDoc (Bio‐Rad) system. The intensities of RNA bands were quantified using ImageLab software.

### Size‐exclusion chromatography coupled with small‐angle X‐ray scattering (SEC‐SAXS)

X‐ray scattering measurements of FASTKΔN1‐75 and FASTKΔN1‐168 were conducted at the B21 beamline of Diamond Light Source (Didcot, UK) [58] using 13.1 keV X‐rays at 50 × 50 μm beam size at the detector with 0.8 × 2 mm photon cross‐section at the sample and flux 4 × 1012 photons/s, and approximately 620 frames (exposure time = 3 s, q range from 0.0045 to 0.34 Å^−1^) were collected per sample using Eiger 4 m detector. Protein samples were separated by the in‐line SEC system (Agilent 1260 HPLC) connected to Superdex 200 Increase 3.2/300 column (GE Healthcare, Chicago, IL, USA) equilibrated in FASTK SAXS Buffer (50 mm Tris/HCl pH 8.5, 5% (v/v) glycerol, 200 mm ammonium sulfate, 50 mm NaCl, 0.5 mm TCEP, 10 mm glutamic acid, 10 mm arginine) using a flow rate of 0.075 mL/min. SEC‐SAXS data processing was performed using the BioXTAS RAW 2.2.1 software package [59, 60] with extension launching ATSAS 3.2.1 software suite [61] using RAW as GUI. The SEC‐SAXS run images were plotted as scattergram (integrated scattering intensity vs. frame number). Buffer regions were selected automatically using liquid chromatography (LC) series analysis tool, and sample regions were selected based on the flatness of Rg parameter. The averaged profiles from buffer‐subtracted images were used for the Guinier analysis. The Guinier fit for FASTK∆N1‐168 data was performed automatically, while FASTK∆N1‐75 data required truncation of the Guinier region to reduce the deviation in the distribution of residuals. The molecular weight of samples was calculated using the following methods: (i) from volume of correlation [62], (ii) from Porod volume [63], (iii) by Shape&Size [64] and (iv) from Bayesian inference method (a part of ATSAS package) [65]. P(r) functions were generated using BIFT algorithm [66]. In both datasets, the qmax values were truncated to 0.1604 in order to obtain reasonable Dmax values and uniform distribution of normalized residuals. The reconstruction of electron density was performed using DENSS program [33] with default settings. All visualizations of protein structures and ED maps were prepared in UCSF Chimera v.1.15 and PyMOL v.2.5.5.

### Footprinting

RNA was 5' end‐labeled with ^32^P using polynucleotide kinase (ThermoFisher Scientific, Waltham, MA, USA) according to the manufacturer's instructions. The labeled RNA was incubated at 37 °C either alone or in the presence of FASTK∆N1‐75 protein in 1× Structure Buffer (Ambion, ThermoFisher Scientific, Waltham, MA, USA), supplemented with 4.3 μm yeast tRNA (Ambion, ThermoFisher Scientific). Reactions were carried out with the addition of either 5 mm lead(II) acetate or 0.001 U·μL^−1^ RNase T1. The reactions were stopped after 2, 4, and 6 min by adding 2× RNA loading dye (Ambion ThermoFisher Scientific). RNA samples were then separated on a polyacrylamide gel containing 7 m urea and analyzed using a Typhoon FLA 9500 scanner. The T1 ladder was prepared by incubating labeled RNA with 0.1 U·μL^−1^ RNase T1 in Sequencing Buffer (Ambion, ThermoFisher Scientific) for 5 min at 37 °C. The alkaline ladder was generated by incubating labeled RNA in Alkaline Hydrolysis Buffer (Ambion, ThermoFisher Scientific) at 95 °C for 5 min.

## Conflict of interest

The authors declare no conflict of interest.

## Author contributions

DMD and MWG designed the study; DMD, KJB, and MWG wrote the first draft of the manuscript; DMD, DAD, MIK, and MK purified proteins; DMD, DAD, and MIK performed RNA‐binding assays; DMD and MIK performed cloning and mutagenesis; DMD performed structural studies and RNA degradation assays; DMD and MMK performed kinase assays; DMD and KJB analyzed degradation assays; KJB performed footprinting assays; MM, DMD, and MWG analyzed structural predictions.

## Supporting information


**Fig. S1.** Thermal shift assays of recombinant FASTK∆N1‐75.
**Fig. S2.** FASTK∆N1‐75 kinase activity assay.
**Fig. S3.** Analysis of GST‐FASTK∆N1‐168 RNA binding specificity by SELEX.
**Fig. S4.** EMSA analysis of FASTK∆N1‐75 binding assays.
**Fig. S5.** Analysis of FASTK∆N1‐75 binding to TERRA RNA.
**Fig. S6.** RNA PAGE of mitochondrial transcripts and footprinting assay.
**Fig. S7.** Analysis of FASTK∆N1‐168 structure.
**Fig. S8.** RNA‐binding of FASTK∆N1‐75 mutants.
**Fig. S9.** Comparison of FASTK and FASTKD4 structures.

**Table S1.** Synthetic RNA oligonucleotides.
**Table S2.** Short RNA transcripts.
**Table S3.** Full‐length RNA transcripts.
**Table S4.** Mass spectrometry analysis of recombinant FASTK samples.
**Table S5.** SAXS data collection and analysis.
**Table S6.** Vectors.
**Table S7.** DNA oligonucleotides.
**Table S8.** DNA starters for generation of the DNA template for *in vitro* transcription.
**Table S9.** Protein sequences of the FASTK recombinant constructs.

## Data Availability

SAXS data were deposited in SASBDB under accession entry identifiers SASDVV2 and SASDVW2.
