# Supplementary material for: Human FASTK preferentially binds single‐stranded and G‐rich RNA
Source: FEBS J. 2025 Jul 1;293(3):729–48. doi: 10.1111/febs.70155 (PMC12871924; doi:10.1111/febs.70155)
Supplement: Supplementary file 1 — Fig. S1. Thermal shift assays of recombinant FASTK∆N1‐75. Fig. S2. FASTK∆N1‐75 kinase activity assay. Fig. S3. Analysis of GST‐FASTK∆N1‐168 RNA binding specificity by SELEX. Fig. S4. EMSA analysis of FASTK∆N1‐75 binding assays. Fig. S5. Analysis of FASTK∆N1‐75 binding to TERRA RNA. Fig. S6. RNA PAGE of mitochondrial transcripts and footprinting assay. Fig. S7. Analysis of FASTK∆N1‐168 structure. Fig. S8. RNA‐binding of FASTK∆N1‐75 mutants. Fig. S9. Comparison of FASTK and FASTKD4 structures. [file FEBS-293-729-s002.pdf]

Human FASTK preferentially binds single-stranded and G-rich RNA

Daria M. Dawidziak[1], Dawid A. Dzadz[1], Mikołaj I. Kuska[1], Madhuri Kanavalli[1], Maria M. Klimecka[1], Matthew Merski[1], Katarzyna J. Bandyra[1], Maria W. Górna[1*]

[1]Structural Biology Group, Biological and Chemical Research Centre, Department of Chemistry, University of Warsaw, Warsaw, Poland

Supplementary Figure 1

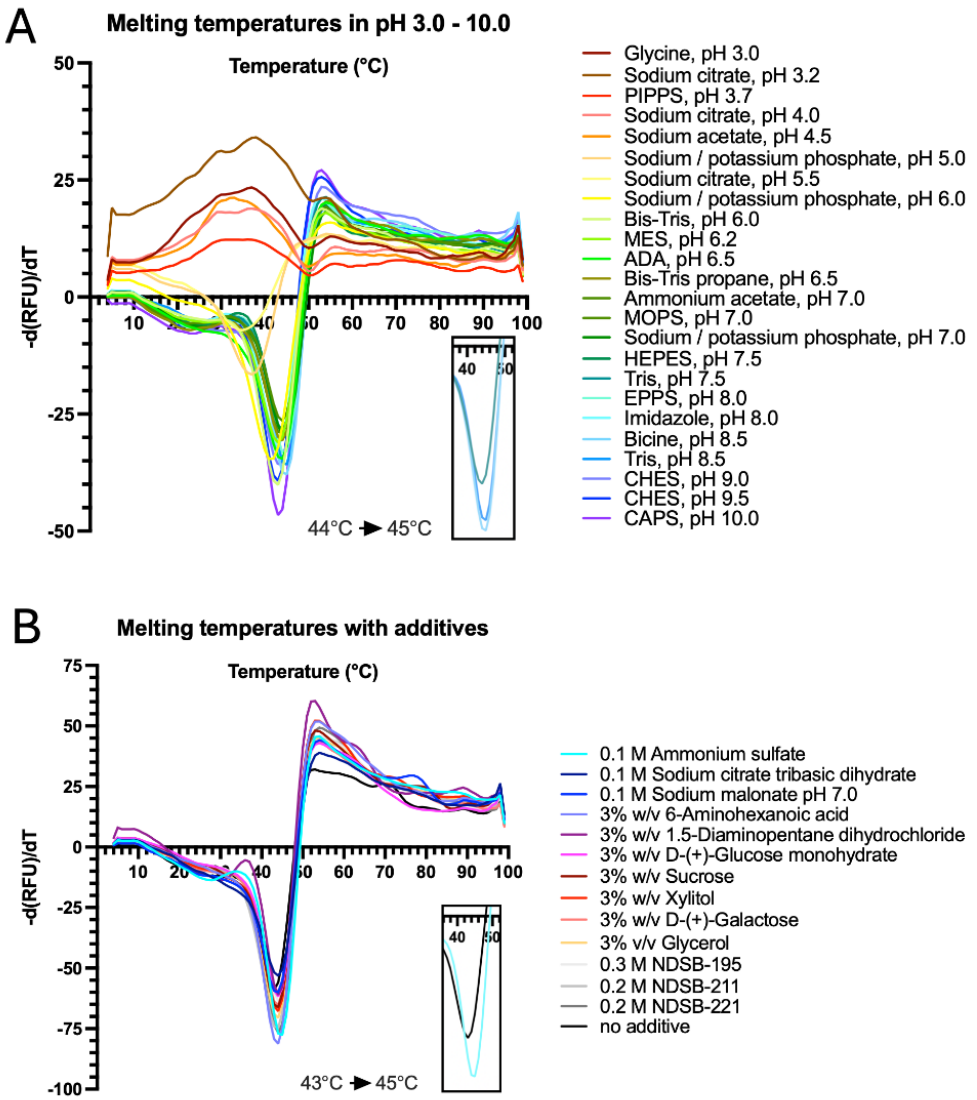

Supplementary Figure 1. A: Thermal shift assay melting curves of FASTKΔN1-75 protein in different pH values. Small box shows a shift in melting temperature of protein in Tris buffer of pH 7.5 (44°C) and protein in Tris or Bicine buffers of pH 8.5 (45°C). B: Thermal shift assay melting curves of FASTKΔN1-75 protein in solutions supplemented with a range of additives. Small box shows a shift in melting temperature of protein in buffer with no additive (43°C) and protein in buffer supplemented with 0.1 M ammonium sulfate (45°C). Due to the limited availability of pure protein at the intitial steps of the project, thermal shift assays were performed once (n = 1).

Supplementary Figure 2

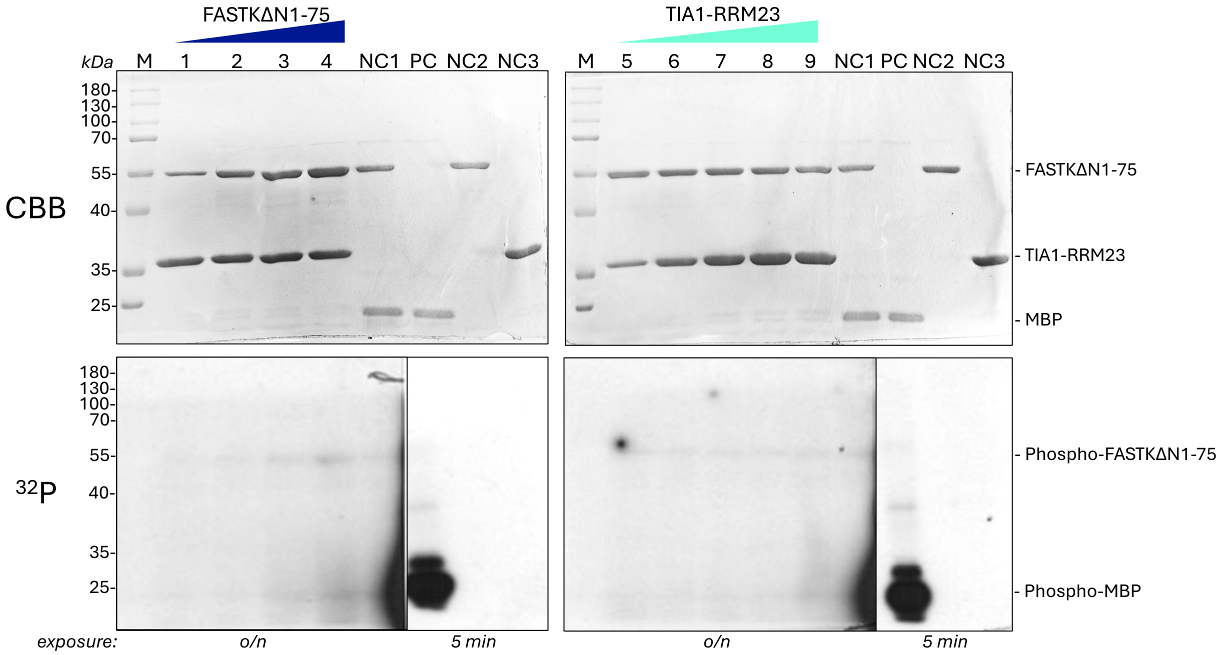

Supplementary Figure 2. The autoradiographs (bottom) and Coomassie Brilliant Blue stained images (top) of kinase activity assay reactions visualized by SDS-PAGE. Left panel shows reactions comprising increasing concentrations of FASTKΔN1-75, while the right panel shows reactions with TIA1-RRM23 concentration gradient. NC – negative control reaction, PC – positive control reaction. Due to limited availability of [γ^{32}P]ATP and control proteins assays were performed once (n = 1).

Supplementary Figure 3

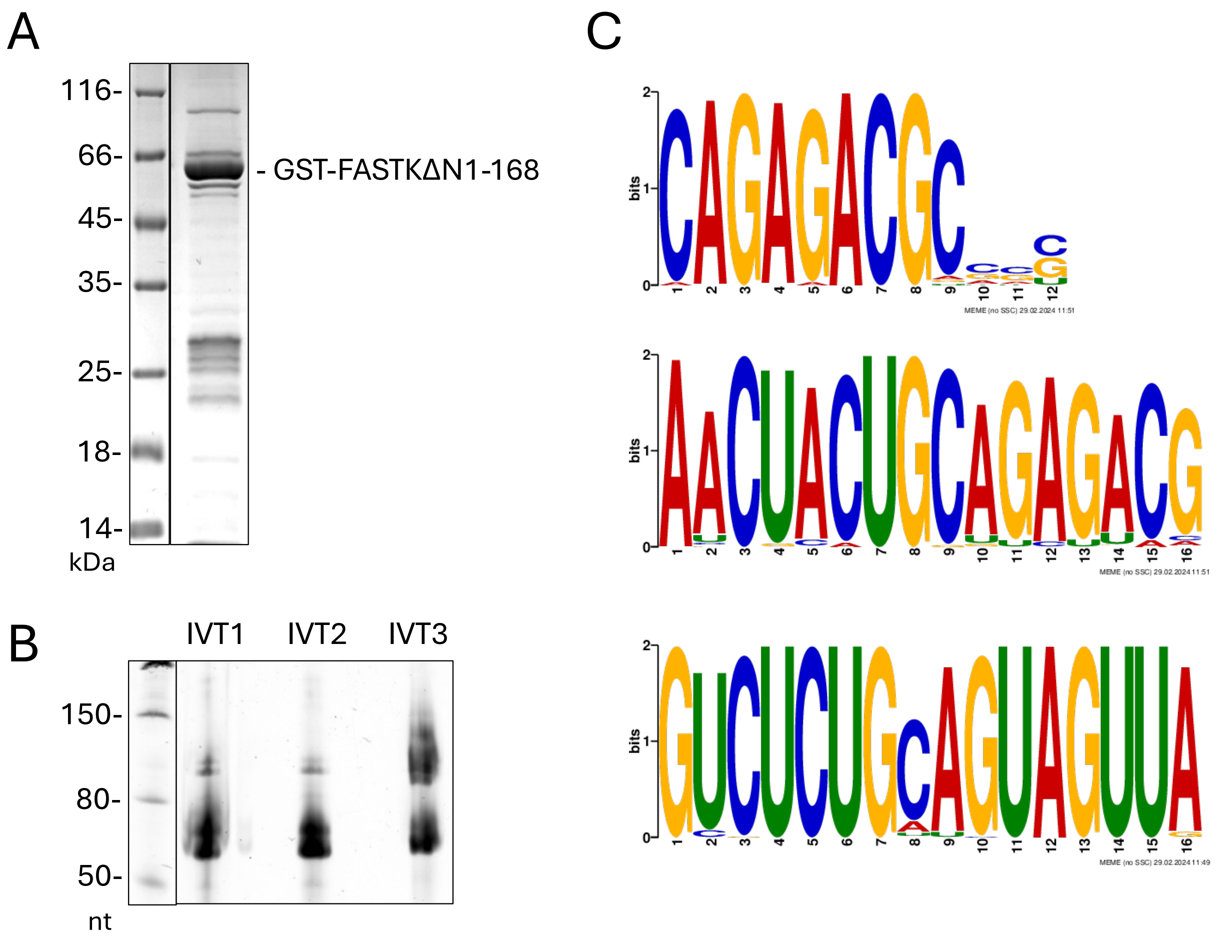

Supplementary Figure 3. Analysis of GST-FASTKΔN1-168 RNA binding specificity by SELEX. A: SDS-PAGE gel of GST-FASTKΔN1-168 protein purified on Glutathione Sepharose column. B: PAGE analysis of RNA oligonucleotides obtained by in vitro transcription reactions (IVT) after each three cycles of SELEX. cDNA amplified in the second cycle was sent for NGS analysis. C: Significant motifs discovered with the motif discovery tool MEME [1,2] detected in 680, 227 and 84 sites, respectively.

Supplementary Figure 4

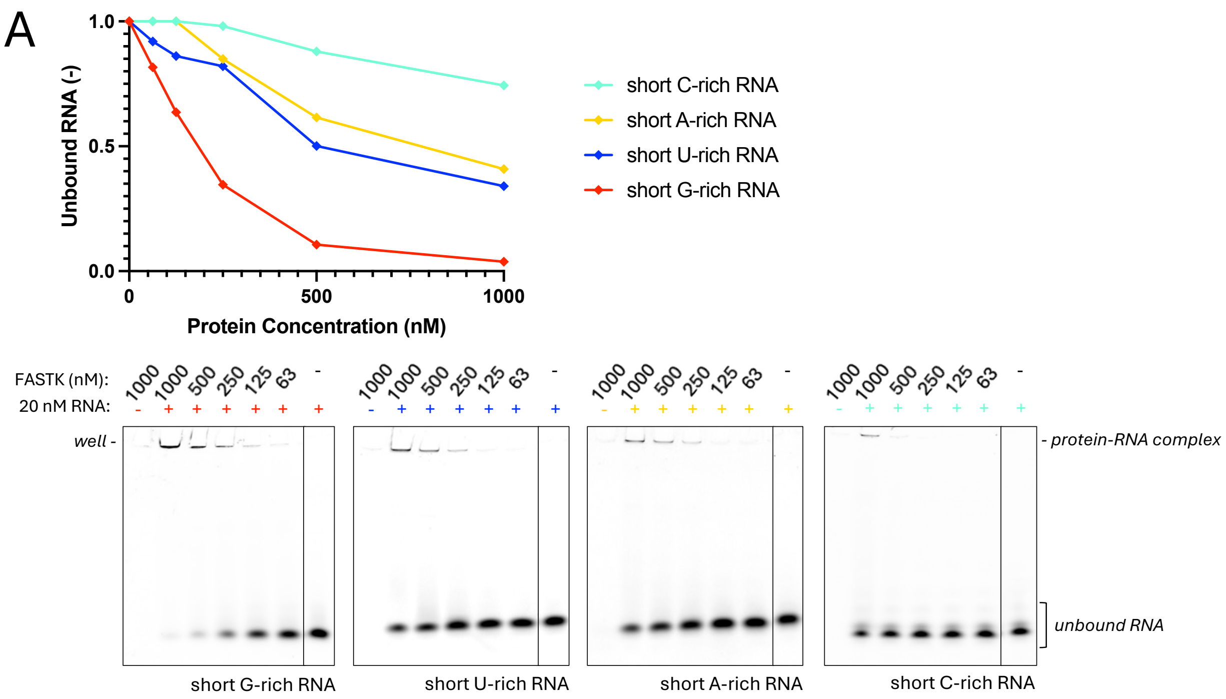

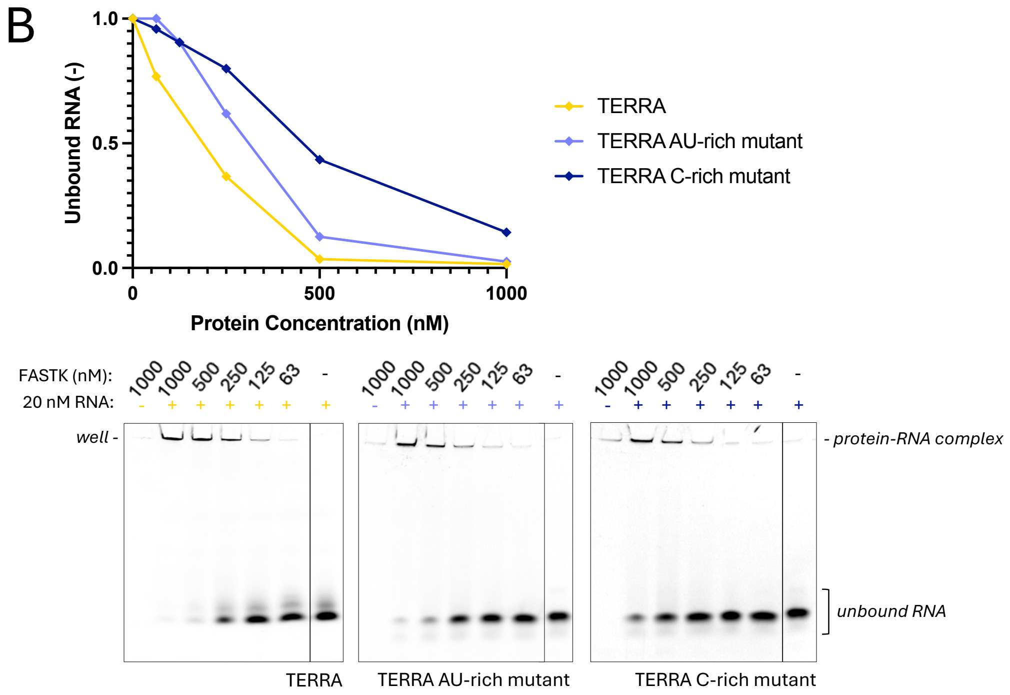

Supplementary Figure 4. EMSA analysis of binding assays of FASTKΔN1-75 with short RNA oligonucleotides (A) and G-quadruplex forming TERRA RNA and its mutants (B). The relative intensities of unbound RNA bands derived from gels scans were plotted against corresponding protein concentrations. Native PAGE gels show shift between protein-RNA complex detained in the gel well and unbound RNA freely migrating in the gel. Due to limited availability of Cy5-labelled RNAs, the EMSA assays were performed once and served to confirm the results previously obtained by MST.

Supplementary Figure 5

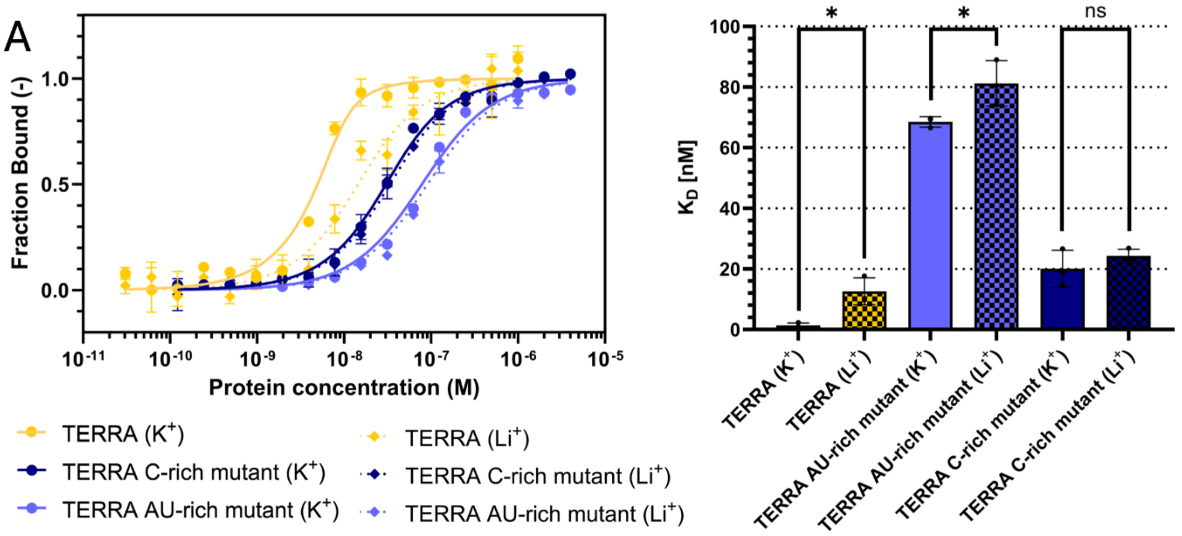

	TERRA (K^+)			TERRA AU-rich mutant (K^+)			TERRA C-rich mutant (K^+)		
	Mean	SD	N	Mean	SD	N	Mean	SD	N
K_D (nM)	1.328	0.844	3	68.486	1.737	3	20.051	6.097	3

	TERRA (Li^+)			TERRA AU-rich mutant (Li^+)			TERRA C-rich mutant (Li^+)		
	Mean	SD	N	Mean	SD	N	Mean	SD	N
K_D (nM)	12.547	4.454	3	81.182	7.516	3	24.315	2.186	3

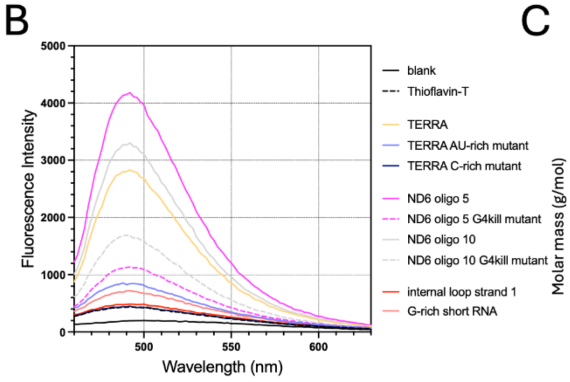

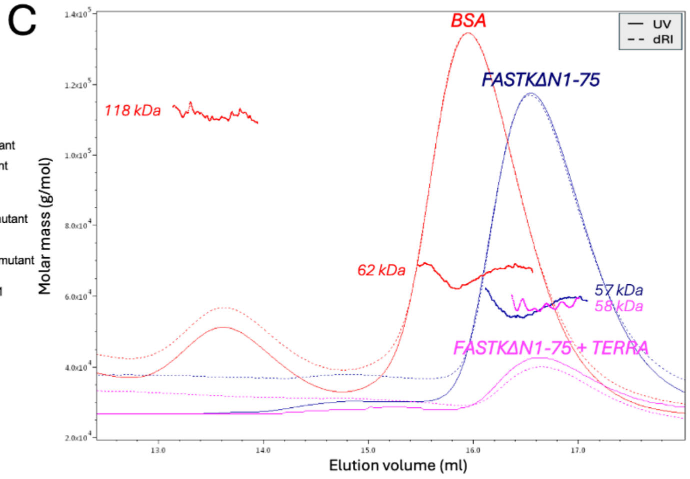

Supplementary Figure 5. A: FASTKΔN1-75 binding assays performed with microscale thermophoresis using G-quadruplex forming TERRA RNA and its mutants in the presence of potassium or lithium ions (and no addition of sodium ions); K_D values were analysed with the two-tailed unpaired parametric t-test, where $p > 0.05$ (ns); $p \leq 0.05$ (*); B: Fluorescence emission spectra of Thioflavin T with chosen RNA oligonucleotides. Due to limited availability of tested RNAs assay was performed once (n = 1). C: Representative results of SEC-MALS analysis of the purified BSA (red trace), FASTKΔN1-75 alone (blue trace) and in complex with TERRA RNA (magenta trace). Curves show the UV absorbance and refractive index signal. Horizontal lines show the calculated masses of the eluting components. BSA and FASTKΔN1-75 were each analyzed in triplicate, but the FASTKΔN1-75–TERRA complex was analyzed in duplicate due to its poor solubility.

Supplementary Figure 6

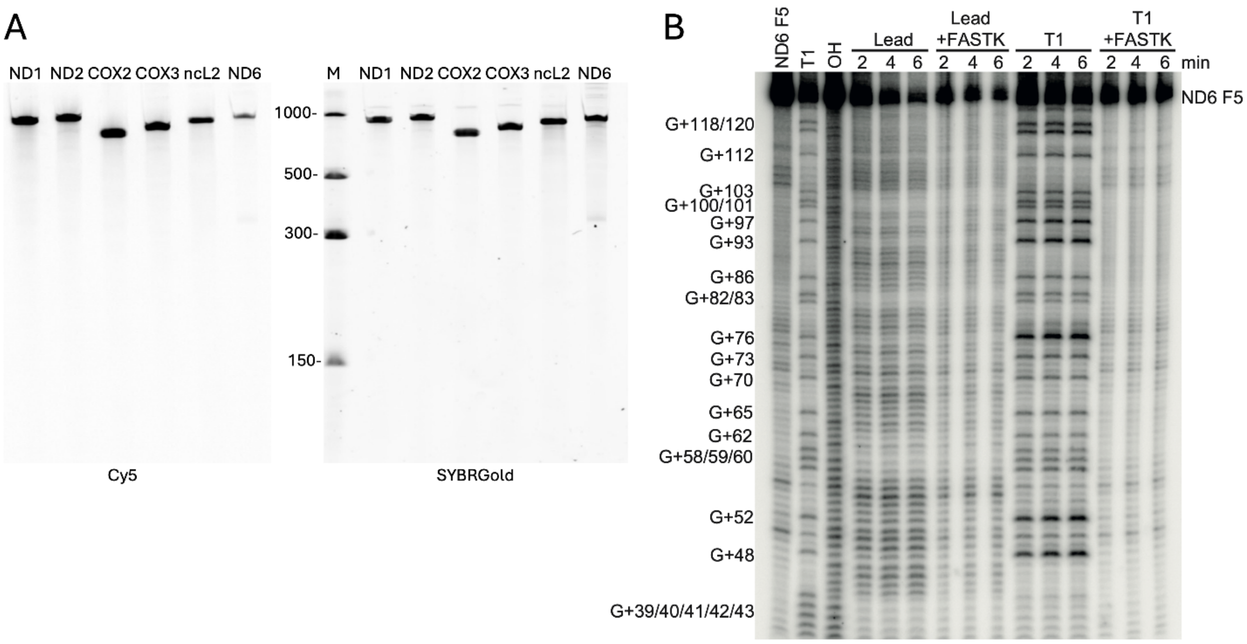

Supplementary Figure 6. A: Representative RNA-PAGE of full-length mitochondrial trascripts used in binding and protection assays. B: Lead and RNase T1 footprinting of ND6 oligo 5 in the absence and presence of FASTKΔN1-75. Due to time constraints, after optimization of reactions which showed similar trends, the final footprinting assay was performed only once.

Supplementary Figure 7

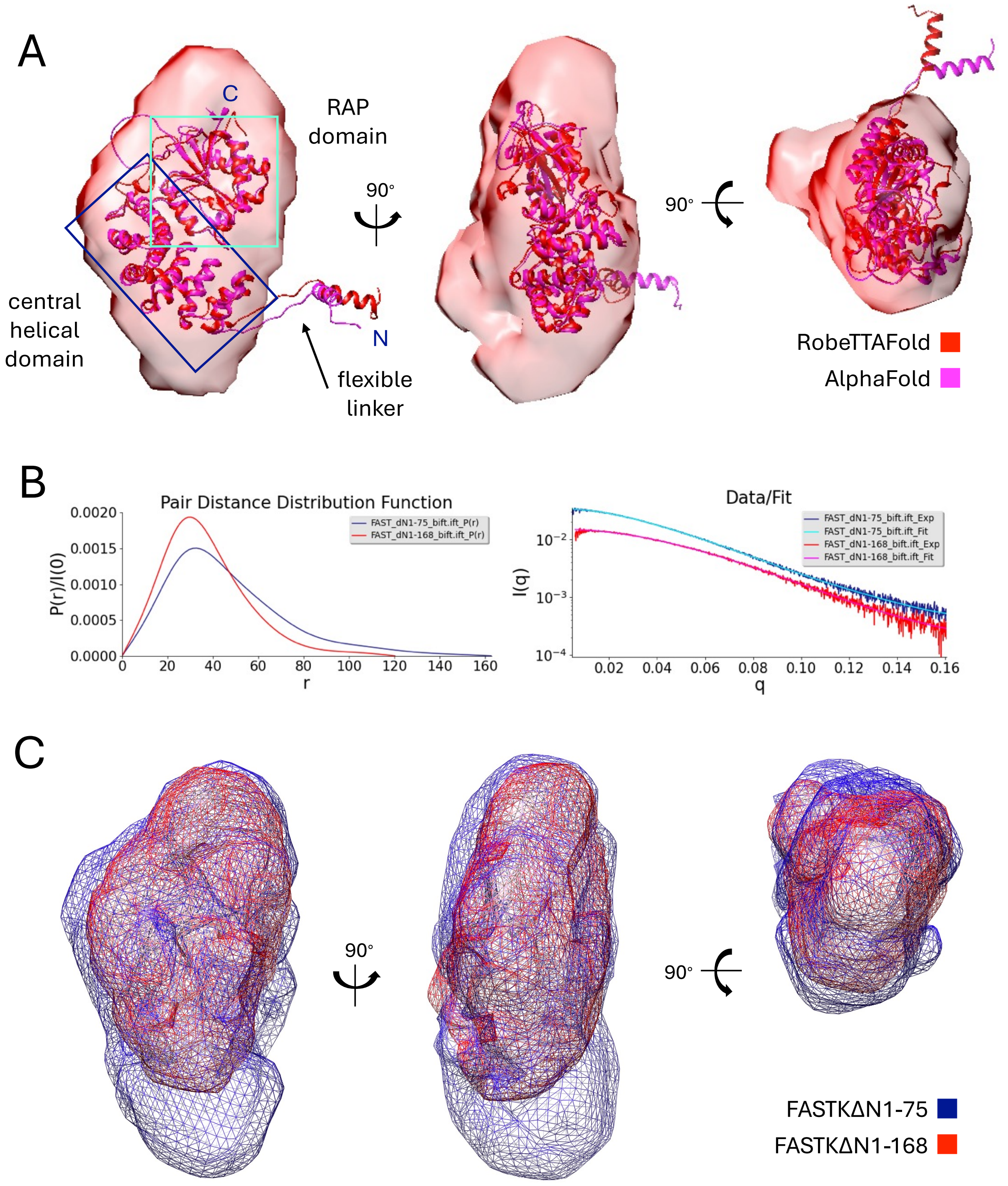

Supplementary Figure 7. A: Front, side, and top views of FASTKΔN1-168 prediction models fitted in the electron density generated by DENSS algorithm [3] based on the SEC-SAXS measurements [4]. Model created with RobeTTAFold [5] (red) was fitted into the map using the Fit in Map tool in UCSF Chimera software, then the AlphaFold model [6,7] (magenta) was aligned to it using the MatchMaker comparison tool. B, left: Pair distance distribution function P(r) calculated for FASTKΔN1-75 (dark blue) and FASTKΔN1-168 (red). B, right: Transformation of the P(r) functions to I(q) fitted to the measured scattering profiles of the corresponding proteins. C: Alignment of FASTKΔN1-75 (purple, mesh) and FASTKΔN1-168 (pink, solid) electron densities generated by DENSS algorithm [3] based on the SEC-SAXS measurements [4].

Supplementary Figure 8

A

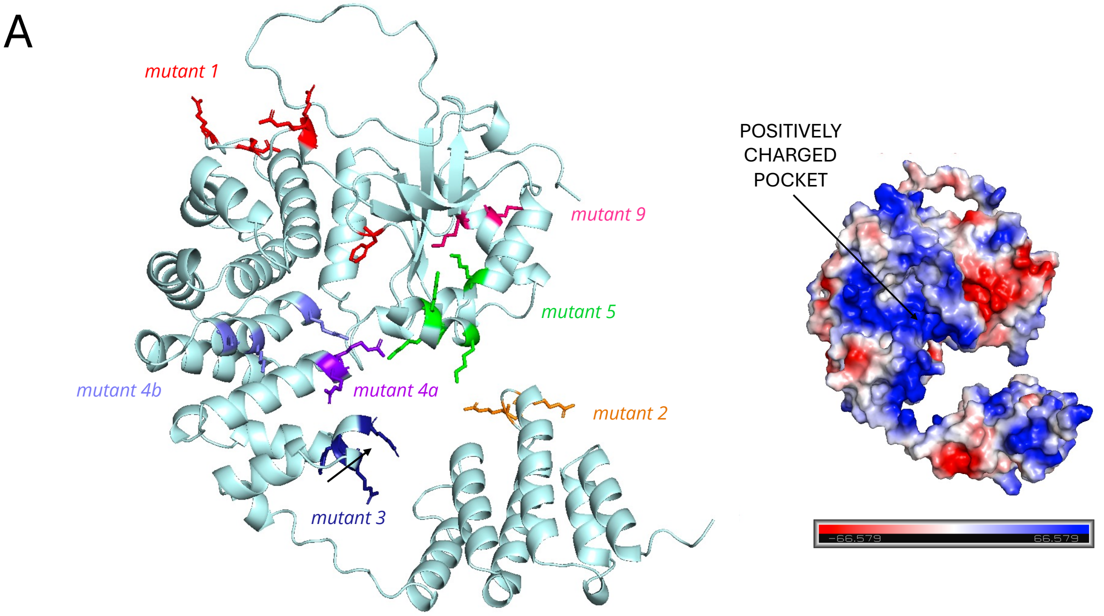

B

Mutant number	1	2	3	4a	4b	5
Mutated residues	R373A, R376A, R379A, R380A, F386A	R128A, R130A	R214A, F215A, R217A	R249A, R251A	K275M, K279M, R286A	R394A, R396A, K398M, K402M
Purification gel	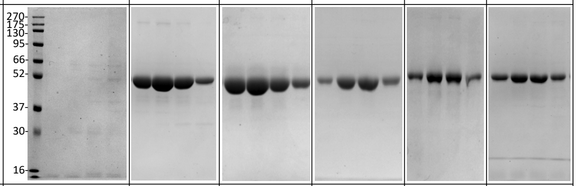					
Contamination with nucleic acids (A_{260}/A_{280})	-	0,63	0,62	0,58	0,60	0,63

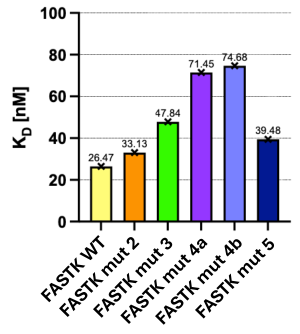

C

Mutant number	6	7	8	9
Mutated residues	R476H	R492H	R501H, R503H	R534H, K536M
Purification gel	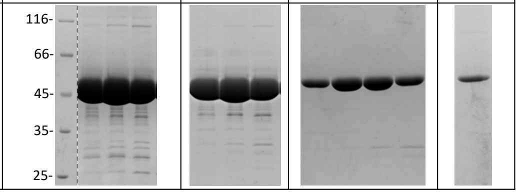			

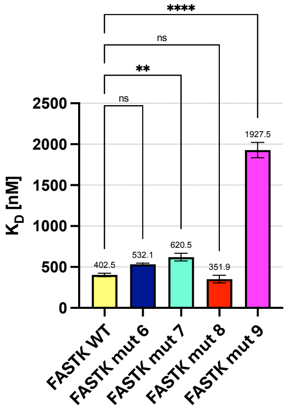

Supplementary Figure 8. A: AlphaFold2 [6] prediction model of FASTKΔN1-75 structure and a map of FASTKΔN1-75 protein contact potential. Highlighted are residues which were modified in each FASTK protein mutant. B: Binding of ND6 oligo 5 RNA by FASTKΔN1-75 and its mutants, determined by microscale thermophoresis. Left panel shows representative SDS-PAGE gels of purified FASTK mutants. Right panel shows K_D values obtained from single measurements (indicated above the bars) and calculated using MO Affinity Analysis software supplied by NanoTemper Technologies. C: Binding of ND6 oligo 5 RNA by FASTKΔN1-75 and its RAP domain mutants, determined by microscale thermophoresis. Left panel shows representative SDS-PAGE gels of purified FASTK mutants. Right panel shows mean K_D values (indicated above the bars) with SD derived from three technical replicates. Statistical significance of pairwise comparisons was calculated with Tukey's multiple comparisons tests for an ordinary one-way analysis of variance (ANOVA): $p > 0.05$ (ns); $p \le 0.05$ (*); $p \le 0.01$ (**); $p \le 0.001$ (***); $p \le 0.0001$ (****). The marker lane, separated by the dashed lane, was taken from another region of the same gel.

Supplementary Figure 9

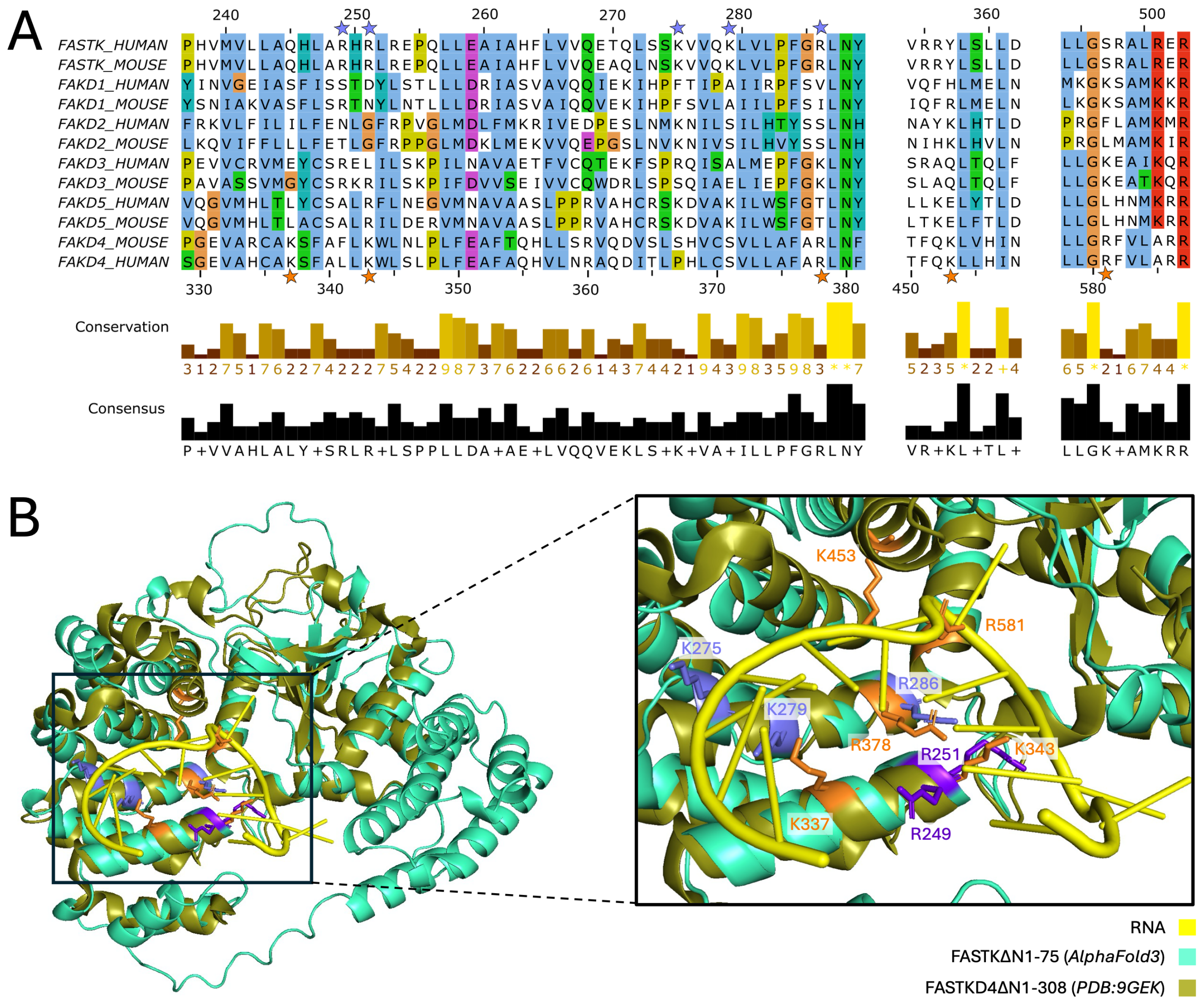

Supplementary Figure 9. A: Multiple sequence aligment (ClustalO [8]) of the central region of FASTKD family members in human and mouse (as indicated by their Uniprot handles), with indicated human FASTK residues R249, R251, K275, K279 and R286 , and human FASTKD4 residues K337, K343, R378, K453 and R581. The panel was prepared with Jalview3 [9] and Inkscape. B: Alignment of the AlphaFold3 [7] predicted structure of the FASTKΔN1-75-G-rich short RNA complex and the experimental structure of FASTKD4 (PDB:9GEK). Residues potentially involved in RNA-binding in FASTK (purple) and FASTKD4 (orange) are highlighted and marked with stars.

References

1 Bailey TL & Elkan C (1994) Fitting a mixture model by expectation maximization to discover motifs in biopolymers. *Proc Int Conf Intell Syst Mol Biol*, 28–36.

2 Bailey TL, Johnson J, Grant CE & Noble WS (2015) The MEME Suite. *Nucleic Acids Res* 43, W39–W49.

3 Grant TD (2018) Ab initio electron density determination directly from solution scattering data. *Nat Methods* 15, 191–193.

4 Cowieson NP, Edwards-Gayle CJC, Inoue K, Khunti NS, Doutch J, Williams E, Daniels S, Preece G, Krumpa NA, Sutter JP, Tully AD, Terrill NJ & Rambo RP (2020) Beamline B21: high-throughput small-angle X-ray scattering at Diamond Light Source. *J Synchrotron Radiat* 27, 1438–1446.

5 Baek M, DiMaio F, Anishchenko I, Dauparas J, Ovchinnikov S, Lee GR, Wang J, Cong Q, Kinch LN, Dustin Schaeffer R, Millán C, Park H, Adams C, Glassman CR, DeGiovanni A, Pereira JH, Rodrigues A V., Van Dijk AA, Ebrecht AC, Opperman DJ, Sagmeister T, Buhlheller C, Pavkov-Keller T, Rathinaswamy MK, Dalwadi U, Yip CK, Burke JE, Christopher Garcia K, Grishin N V., Adams PD, Read RJ & Baker D (2021) Accurate prediction of protein structures and interactions using a three-track neural network. *Science (1979)* 373, 871–876.

6 Jumper J, Evans R, Pritzel A, Green T, Figurnov M, Ronneberger O, Tunyasuvunakool K, Bates R, Žídek A, Potapenko A, Bridgland A, Meyer C, Kohl SAA, Ballard AJ, Cowie A, Romera-Paredes B, Nikolov S, Jain R, Adler J, Back T, Petersen S, Reiman D, Clancy E, Zielinski M, Steinegger M, Pacholska M, Berghammer T, Bodenstein S, Silver D, Vinyals O, Senior AW, Kavukcuoglu K, Kohli P & Hassabis D (2021) Highly accurate protein structure prediction with AlphaFold. *Nature* 596, 583–589.

7 Abramson J, Adler J, Dunger J, Evans R, Green T, Pritzel A, Ronneberger O, Willmore L, Ballard AJ, Bambrick J, Bodenstein SW, Evans DA, Hung C-C, O'Neill M, Reiman D, Tunyasuvunakool K, Wu Z, Žemgulytė A, Arvaniti E, Beattie C, Bertolli O, Bridgland A, Cherepanov A, Congreve M, Cowen-Rivers AI, Cowie A, Figurnov M, Fuchs FB, Gladman H, Jain R, Khan YA, Low CMR, Perlin K, Potapenko A, Savy P, Singh S, Stecula A, Thillaisundaram A, Tong C, Yakneen S, Zhong ED, Zielinski M, Žídek A, Bapst V, Kohli P, Jaderberg M, Hassabis D & Jumper JM (2024) Accurate structure prediction of biomolecular interactions with AlphaFold 3. *Nature* 630, 493–500.

8 Madeira F, Madhusoodanan N, Lee J, Eusebi A, Niewielska A, Tivey ARN, Lopez R & Butcher S (2024) The EMBL-EBI Job Dispatcher sequence analysis tools framework in 2024. *Nucleic Acids Res* 52, W521–W525.

9 Waterhouse AM, Procter JB, Martin DMA, Clamp M & Barton GJ (2009) Jalview Version 2—a multiple sequence alignment editor and analysis workbench. *Bioinformatics* 25, 1189–1191.
